# ImmunoPepper: extracting personalized peptides from complex splicing graphs

**DOI:** 10.1093/bioinformatics/btaf492

**Published:** 2025-10-09

**Authors:** Laurie Prélot, Jiayu Chen, Matthias Hüser, André Kahles, Gunnar Rätsch

**Affiliations:** Department of Computer Science, ETH Zürich, Zürich 8092, Switzerland; Biomedical Informatics Research, University Hospital Zürich, Zürich 8006, Switzerland; SIB Swiss Institute of Bioinformatics, Zürich 8057, Switzerland; Department of Computer Science, ETH Zürich, Zürich 8092, Switzerland; Department of Computer Science, ETH Zürich, Zürich 8092, Switzerland; SIB Swiss Institute of Bioinformatics, Zürich 8057, Switzerland; Department of Computer Science, ETH Zürich, Zürich 8092, Switzerland; Biomedical Informatics Research, University Hospital Zürich, Zürich 8006, Switzerland; SIB Swiss Institute of Bioinformatics, Zürich 8057, Switzerland; Department of Computer Science, ETH Zürich, Zürich 8092, Switzerland; Biomedical Informatics Research, University Hospital Zürich, Zürich 8006, Switzerland; SIB Swiss Institute of Bioinformatics, Zürich 8057, Switzerland; Department of Biology, ETH Zürich, Zürich 8093, Switzerland; ETH AI Center, Zürich 8092, Switzerland

## Abstract

**Motivation:**

RNA sequencing enables the characterization of a cell’s transcript isoforms in healthy and disease conditions. In the context of cancer, local transcript variability may translate to splicing-derived tumor-associated peptides recognized by the immune system. A software tool that extracts such candidate peptides, is of great interest for personalized cancer therapy.

**Results:**

We present the open-source software tool *ImmunoPepper*, which extracts a set of biologically plausible peptides from a splicing graph, derived from a set of RNA-seq datasets. This peptide set can be personalized with germline and somatic variation and takes novel RNA splice variants into account. *ImmunoPepper* supports several filtering options, including subtraction of normal tissue background, prediction of MHC-binding affinity, as well as MassSpec-based validation of identified peptides. We analyzed 32 ovarian cancer (TCGA-OV) and 31 breast invasive carcinoma (TCGA-BRCA) samples, with a strict cancer-specific filtering configuration, and obtained on average 834 and 569 cancer-specific predicted MHC-I binding 9-mers per sample, for each cohort, respectively. MassSpec validation with the target-decoy competition *Subset-Neighbor-Search* (*SNS*) showed an average validation rate of 4.5% per TCGA-OV sample and 5.3% per TCGA-BRCA sample. This corresponded to 25 MHC-I binders 9-mers per TCGA-OV sample, and 20 MHC-I binders 9-mers per TCGA-BRCA sample in average. Finally, we draw conclusions about the best framework for generation of splicing-derived neoepitopes and recommend to use joint data structures when processing homogeneously a cancer and a normal cohort and to focus on reproducibility of the candidates across generation pipelines.

**Availability and implementation:**

*ImmunoPepper* is implemented in Python 3 and is available as open-source software at https://github.com/ratschlab/immunopepper. The online documentation can be found at https://immunopepper.readthedocs.io/en/latest/.

## 1 Introduction

High-throughput RNA sequencing (RNA-seq) is a well-established technique to measure a cell’s transcriptome. It is often used as a proxy for assessing quantitative features such as gene expression. The prediction of qualitative features, for instance, protein isoforms and variants, is gaining importance for personalized cancer vaccine design (Guo *et al.* 2013). Identifying tumor antigens—short tumor-associated peptides that can be recognized by the immune system on the outer surface of tumor cells—is one of the pre-requisites of personalized cancer vaccine development and involves a multi-step process (Richters *et al.* 2019).

In earlier works, the prediction of such peptides only considers recurrent non-synonymous single nucleotide variants (SNVs) that introduce coding changes in the amino-acid sequence (Schumacher and Schreiber 2015), (Bjerregaard *et al.* 2017, Tappeiner *et al.* 2017, Zhang *et al.* 2017, Kim *et al.* 2018, Hundal *et al.* 2020, Wood *et al.* 2020) and other variants like INDELs (Wang *et al.* 2019) or gene fusion (Zhang *et al.* 2017, Wei *et al.* 2019). Other alteration paths, such as alternative transcript processing or alternative splicing, have also been explored for their potential to create neoepitope candidates (Kahles *et al.* 2018, Smart *et al.* 2018). While several software tools have recently been developed to extract splicing-derived neoepitope candidates (Zhang *et al.* 2020, Chai *et al.* 2022, Pan *et al.* 2023, Schäfer *et al.* 2023, Li *et al.* 2024), so far, no software tool is available to predict such peptides derived from alternative exon and intron usage in a personalized manner, i.e. to take germline and somatic mutations of a patient sample into account together with splicing variants on the same peptide. Previous efforts in this direction are Neoepiscope (Wood *et al.* 2020), which models the co-occurrence of germline variants and somatic mutations but not splicing variation, Neosplice (Chai *et al.* 2022) which extracts either splicing- or somatic mutation-derived neoepitope candidates, or Splice2neo (Lang *et al.* 2024), RegTools (Cotto *et al.* 2023) and SPLICE-neo (Wickland *et al.* 2024) which focus on splicing caused by somatic mutations. Developments in the field of multi-alterations-derived neoepitope candidates include the tool presented in this article, *ImmunoPepper*, and a parallel work presented in (Zhu *et al.* 2024). Notably, few tools in the past have leveraged the expressivity of a splicing graph for this task (Chai *et al.* 2022, Zhu *et al.* 2024) with most methods relying on the insertion of RNA-seq supported junctions into annotated linear transcripts (Zhang *et al.* 2020, Pan *et al.* 2023).

Mass spectrometry (MS) has been presented in the past as a high-throughput *in-silico* validation method for extracted peptide candidates (Hu *et al.* 2016, Kahles *et al.* 2018, Zhang *et al.* 2019). Neoepitope analyses have also benefited from the emergence of MHC immunoprecipitation and subsequent MS (Laumont *et al.* 2018, Chong *et al.* 2020); this approach has been named *immunopeptidomics* or *ligandomics* and aims at characterizing the *immunopeptidome*. Both techniques have been demonstrated to validate splicing-derived neoepitope candidates (Zhang *et al.* 2020, Chai *et al.* 2022, Pan *et al.* 2023, Li *et al.* 2024). However, seamless integration into a software tool for prediction of splicing-derived neoepitope candidates has to our knowledge only been realized by the IRIS software tool (Pan *et al.* 2023).

*ImmunoPepper* aims to serve this need and provide a versatile, modular, and integrated tool addressing the automatic extraction of personalized, splicing-derived putative neoepitopes from RNA-seq data. To achieve this goal, *ImmunoPepper* translates peptides from a given splicing graph through a propagation of the reading frame across the graph’s paths. *ImmunoPepper* allows filtering of these candidates by expression, MHC-binding, and mass spectrometry validation criteria. In Table 3, available as supplementary data at *Bioinformatics* online, we compare *ImmunoPepper* to other software tools with respect to features that enrich the pool of detected putative neoepitopes. Detailed comparison of the features of *ImmunoPepper* can be found in Table 4, available as supplementary data at *Bioinformatics* online.

In the following, we describe *ImmunoPepper’*s features, study its validation rate across multiple MS analysis methods, and reproduce earlier results from (Kahles *et al.* 2018), a previous analysis showing the potential of splicing-derived neoantigens.

## 2 The ImmunoPepper algorithm for generating personalized peptides from splice graphs

### 2.1 Preliminaries and notation

The peptide extraction algorithm of *ImmunoPepper* takes as input a so-called splicing graph built from an annotation file in General Feature or General Transfer format (GFF/GTF) and augmented with RNA-seq data. The splicing graph is traversed, generating a set of peptide fragments, that are later filtered to yield putative neoepitopes.

We assume that a gene G has *k* different transcripts $t_{1},t_{2},\ldots ,t_{k}\in T_{G}$, where $T_{G}$ is the set of all transcripts of the gene of interest.

We define a splicing graph as a directed acyclic graph G=(V,E) where the exon set *V* is the set of exons occurring in a given set of transcripts and the edge set *E* are the connections between exons, representing introns, i.e. possibilities of alternative splicing.

An exon is given by $v=(\text{start},\text{end})=(g_{v_{\text{start}}},g_{v_{\text{end}}})\in N^{2}$ where $g_{v_{\text{start}}}$ and $g_{v_{\text{end}}}$ is the pair of genomic coordinates associated with the genomic interval $[g_{v_{\text{start}}},g_{v_{\text{end}}}[$.

Transcripts are sets of exons connected by introns. Transcripts have a direction, and each exon is assigned an index that follows the sorted order of the exons in the transcript. The exon set $V_{i}=\{v_{i,1},\ldots ,v_{i,d_{i}}\},1\leq i\leq k$ associated with the transcript $t_{i}$ is constituted of its exons and $d_{i}$ is the total number of exons in the transcript. The set of exons occurring in any transcript $t_{i}$ of the gene G is denoted by *V*,


$$V:=\overset{k}{\underset{i=1}{\cup }}V_{i}$$

and the set of connections occurring in any transcript $t_{i}$ of the gene G is denoted by *E*


$$E:=\overset{k}{\underset{i=1}{\cup }}E_{i}\subseteq V\times V.$$

$E_{i}$ are the edges implied by transcript $t_{i}$ and *k* is the total number of transcripts in the gene from which the splicing graph is constructed.

**Figure 1.**
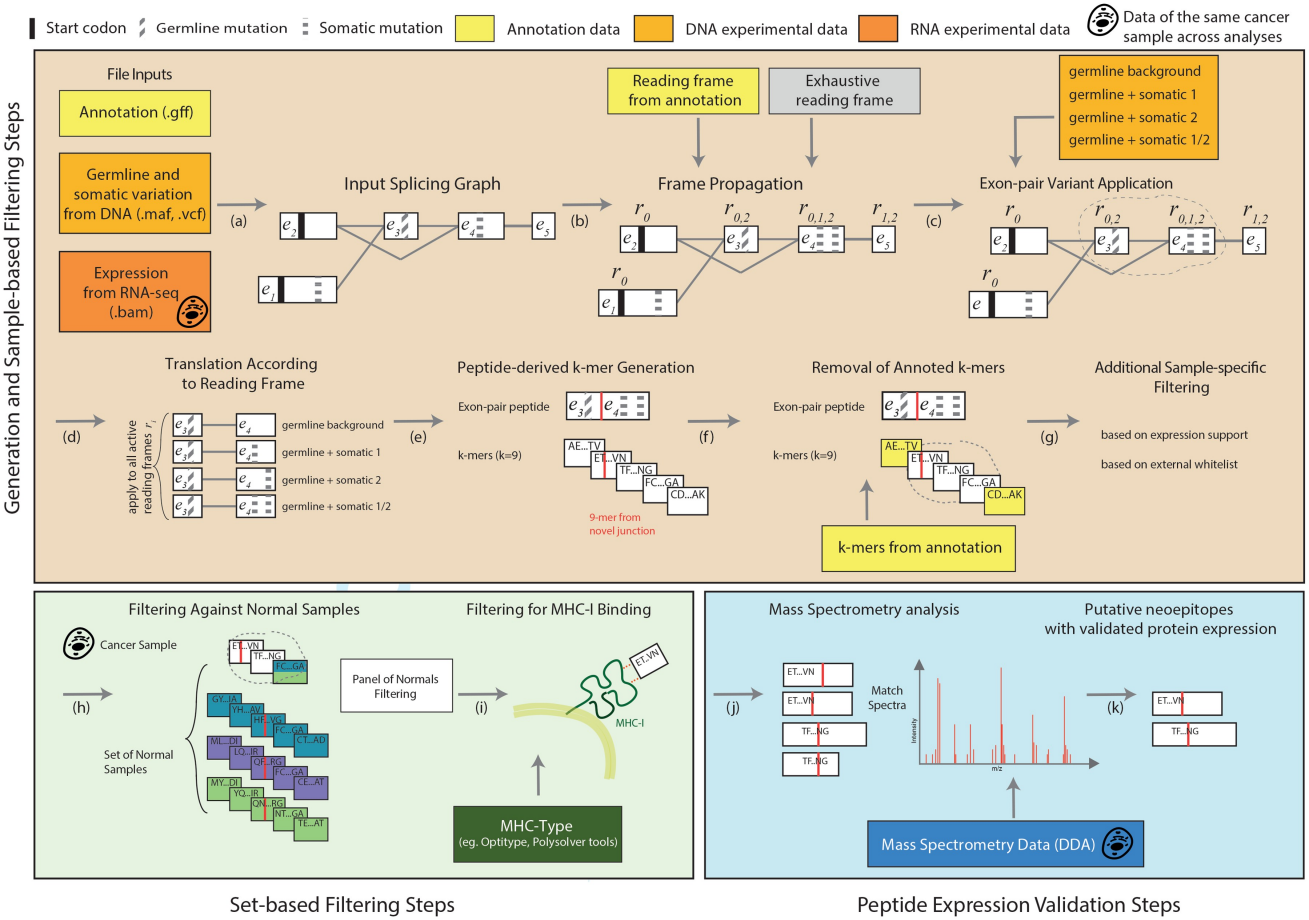
Overview of the main steps of *ImmunoPepper*. (a) Inputs to the workflow are an annotation file, variant calls (optional) and aligned RNA-seq reads. (b) A splicing graph is generated with SplAdder (Kahles *et al.* 2016) with exons depicted as black boxes and introns as gray lines. The splicing graph is processed with *Immunopepper* and annotated with translation start sites (TSS, black bars) as well as germline (horizontally striped bars) and somatic (diagonally striped bars) variants. Exons are numbered from $e_{1}$ to $e_{5}$. (b and c) Reading frames are propagated from the TSS across the edges of the graph. Each exon is labeled with the possible reading frame offsets that can occur during translation. (d) Each exon pair is then translated into peptide sequences, taking personal genome variation into account and allowing for combinations of somatic variants. (e) Generated peptides are split into *k*-mers, with *k* specified by the user. (f) *k*-mers which are present in the annotation file are not considered as candidates and filtered out. (g) *k*-mer candidates from each sample can be selected based on additional properties, such as their expression support in the sample or their presence in a whitelist. (h) Tumor-associated candidates are selected (they are found in cancer samples only, or in cancer samples and low presence in normal samples). (i) Assessment of MHC-I binding properties of the *k*-mers is performed. (j) The filtered *k*-mers are mapped back to their longer context peptides (two or three exons). The peptides can be digested with a restriction enzyme such as trypsin and their protein-level expression can be assessed in input Data-dependent acquisition (DDA) bottom-up proteomics data. (k) The final set of candidate peptides is generated. All steps (f–k) are optional. The software can also be run without the personalization displayed in (c).

A splicing graph can be pre-computed using the external software *SplAdder* (Kahles *et al.* 2016), and then provided to *ImmunoPepper* (Fig. 1a).

### 2.2 Anchoring translation start positions and initial reading frames

A genome annotation provided as input contains all known transcripts and their associated protein coding sequences (CDS), also known as coding regions. A CDS region is represented as a coordinate pair $c:(g_{c_{\text{start}}},g_{c_{\text{end}}})$. CDS regions overlap exon coordinates so that CDS start and ends must be located on an exon. Each transcript $t_{k}$ contains of a set of CDS regions $t_{k}:=\{c_{k,1},c_{k,2}\ldots ,c_{k,m_{k}}\}$, where $m_{k}$ is the number of CDS regions for transcript $t_{k}$. The indexes $1,2,\ldots ,m_{k}$ follow the sorted order of the CDS regions in the transcript $t_{k}$.

In some cases, the translation does not start from the beginning of an exon and hence only a part of it is identified with the translation start CDS. For each CDS region starting a translation, we extract the initial set of *reading frames* ⊆{0,1,2} from the given reference annotation, which are defined as the possible offsets for translation initiation. We denote the seed reading frame(s) for transcript $t_{k}$ as $R_{k}$ (technically, if the start coordinates are adapted accordingly, the initial reading frame for all translation start sites can be 0). Any transcript $t_{k}$ to be translated needs two pieces of information: the translation start site $g_{t_{k_{\;\text{start}}}}$ and the seed reading frames $R_{k}$ which are stored in the translation tuple $\left(g_{t_{k_{\;\text{start}}}},R_{k}\right)$. On the positive strand $g_{t_{k_{\text{start}}}}=g_{c_{k,1_{\;\text{start}}}}$, where $g_{c_{k,1_{\;\text{start}}}}$ marks the start position of the CDS region $c_{k,1}$. On the negative strand $g_{t_{k_{\;\text{start}}}}=g_{c_{k,m_{k}\;\text{end}}}$ where $g_{c_{k,m_{k}\;\text{end}}}$ marks the end position of the CDS region $c_{k,m_{k}}$. In general, there can be multiple translation start sites annotated for a given splicing graph, resulting in multiple possible reading frame initiations.

To assign the initial reading frame sets, we identify each translation tuple $\left(g_{t_{k_{\text{start}}}},R_{k}\right)$ with all splicing graph exons *v* such that $g_{v_{\text{start}}}\leq g_{t_{k_{\text{start}}}}\leq g_{v_{\text{end}}}$ and assign the implied initial reading frames to the exon. Each exon $v_{i}$ can have assigned at most three initial reading frames ∈{0,1,2} at the end of this step, defining an exon, read-frames tuple $\left(v_{i},R_{i}\right)$.

### 2.3 Splice graph traversal and translation

In the following, we describe the translation from reading frames implied by a given annotation file. First, we collect all coding start positions and reading frames across all known transcripts from the file (Fig. 1b). We traverse the graph taking into account frameshifts. Starting from the anchored translation start CDS regions: for an exon $\left(v_{i},R_{i}\right)$, we consider the exons connected with and following $v_{i}$ in read strand order, that is $VF_{i}:\{v_{j}|\;(v_{i},v_{j})\in E\wedge j>i\}$. Then, we translate any two exons connected by an edge (as well as any exons without any incoming or outgoing edges, which we refer to as *isolated* exons): For each exon $v_{i}$ and each directly following exon $v_{j}\in VF_{i}$, and each $r\in R_{i}$, we translate the sequence $Q:=(g_{v_{i_{\text{start}}}}+r,g_{v_{i_{\text{end}}}})$. Define $\text{res}:=(g_{v_{i_{\text{end}}}}-g_{v_{i_{\text{start}}}}-r)\;\mathrm{mod}\;3$ and $r^{\prime }=(3-\text{res})$. Hereby res is the number of bases left and $r^{\prime }$ is the number of residual bases needed to borrow from $v_{j}$ so that we can translate a full amino acid. When translating $v_{j}$, we start from $g_{v_{j_{\text{start}}}}+r^{\prime }$. In other words, $r^{\prime }$ is the reading frame to be propagated to $v_{j}$. In the traversal process, we iterate over all connected exon pairs $\left(v_{i},v_{j}\right)$ in ascending topological (or descending, according to the read strand) order and propagate all active reading frames R from $v_{i}$ to $v_{j}$: After translation of $Q,v_{j}$ will be updated with the propagated reading frame to yield the new exon state $\left(v_{j},R_{j}^{\prime }\right)$ where $R_{j}^{\prime }\leftarrow R_{j}\cup \{r^{\prime }\}$. If there is a stop codon when translating pair $\left(v_{i},v_{j}\right)$ for some r, the corresponding reading frame $\left\{r^{\prime }\right\}$ will not be propagated to $v_{j}$, as the translation is truncated. For simplicity, the above description assumes the positive strand case, the negative strand works analogously, but in the reverse way. The translation with *ImmunoPepper* can also be performed similarly but considering all three possible reading frames for translation.

### 2.4 Personalization of translated sequences with personal variation

For the personalization of splice variant sequences generated from exon pairs, we consider given somatic mutations from one cancer sample in any possible combination of the transcript. For the germline variants, *ImmunoPepper* currently considers the cases where all germline variants are homozygous, or all variants are heterozygous on the same allele, meaning that they are all applied jointly, or are all absent (reference). Specifically, all germline variants are applied jointly and map the original gene sequence g to $g^{GM}$. Performing the previously described traversal with respect to $g^{GM}$ then yields the peptides personalized with germline variants. In contrast, for somatic mutations, we generate all possible combinations of local somatic mutations one by one, on the fly, as we process a particular exon pair of the splicing graph. For exon pair $\left(v_{i},v_{j}\right)$, define $SM_{i,j}$ as the mutation set containing all somatic mutations whose positions are in $[g_{v_{i_{\text{start}}}},g_{v_{j_{\text{end}}}}[$. If $|SM_{i,j}|=n$. There are $2^{n}$ combinations of somatic mutations and thus $2^{n}$ different sequences will be generated. *ImmunoPepper* can integrate both somatic mutations and germline variation, germline or somatic only, or no personal variation at all (Fig. 1c). For higher sensitivity, one should use the union of all these settings. To model more completely the mutational processes, *ImmunoPepper* would take as input the phased germline variants and apply them together with any combination of somatic mutations.

Redundant sequences from the peptide output are excluded. For two exon pairs $\left(v_{i},v_{j}\right)$, $\left(v_{s},v_{t}\right)$ with $v_{i}<v_{j}$ and $v_{s}<v_{t}$, if $v_{i_{\text{start}}}\geq v_{s_{\text{start}}}\wedge v_{i_{\text{end}}}=v_{s_{\text{end}}}\wedge v_{j_{\text{start}}}=v_{t_{\text{start}}}\wedge v_{j_{\text{end}}}\leq v_{t_{\text{end}}}$ and they have the same reading frames R, the peptide set translated from $\left(v_{i},v_{j}\right)$ will be a subset of the one translated from $\left(v_{s},v_{t}\right)$. To reduce redundancy, we do not output the peptides generated by $\left(v_{i},v_{j}\right)$.

### 2.5 Generation of *k*-mers from longer multi-exon peptide sequences

For each peptide of length L, all possible *k*-mers are generated, which represents at most L−k+1 distinct *k*-mers. If an exon pair $\left(v_{i},v_{j}\right)$ is relatively short, i.e. if $|v_{i}+v_{j}|<3k$, then potential candidates *k*-mers cannot be generated from the pair and information is potentially lost. In this case, we consider exon-triplets instead of exon pairs when generating *k*-mers from the sequences. A 4-exon peptide would only be necessary if the cumulative exon length of three subsequent nodes in the splicing graph is still shorter than *k*. Based on this observation, we use triplets and found 0.5% to 4% peptides coming from triplets in TCGA-BRCA and TCGA-OV cohorts. However, there is no technical limitation and quadruplets or an iterative addition of exons could be performed.

## 3 ImmunoPepper: a novel software tool with integrated peptide filtering and validation

### 3.1 Capabilities of the software tool

#### 3.1.1 Generation of candidate peptides

*ImmunoPepper* allows the generation of splicing-derived neoantigens, with or without personalization with germline variants or somatic mutations. The generation algorithm is described in detail in Section 2. It takes as input a splice graph generated with *SplAdder* (Kahles *et al.* 2016) as well as an annotation file in GFF/GTF format and a reference genome in FASTA format (Fig. 1a). Connected exon pairs are translated taking into account frameshifts (Fig. 1d). Additional exon-triplets are translated when the desired *k*-mer length cannot be solely achieved with exon pairs. It outputs 2- and 3-exon peptides and all sub-strings of length k derived from the 2- and 3-exon peptides (Fig. 1e). This output set can be generated on a disease cohort (e.g. cancer) or a normal cohort. For downstream analyses, these sets constitute the *foreground* set of potential candidates, and the *background* set of normal peptides, respectively. The software also outputs multi-exon peptides for all transcripts present in the annotation. This set constitutes the set of *annotated* peptides. The *annotated* peptides are generally included in the *background* set because they do not constitute novelty specific to the patient cohort.

The 2- and 3-exon peptides have associated metadata, which includes the coordinates from which the peptide is derived, the annotation status of the junction, the reading frame applied, and whether the translation of the last exon was interrupted by a stop codon. The candidate *k*-mers’ metadata includes the expression as quantified by RNA-seq reads overlapping the genomic positions of the *k*-mer’s or, if the *k*-mer includes more than one exon, the quantification from reads overlapping the genomic positions of the junction(s) (Supplementary Methods, available as supplementary data at *Bioinformatics* online).

The generation can be performed by enabling on-the-fly exclusion of *k*-mers present in a user-provided database. This allows the user to spend computational time only on generating outputs restricted to *k*-mers of interest.

#### 3.1.2 Filtering of candidate peptides based on a foreground cancer cohort and a background normal cohort

*ImmunoPepper* provides flexible filtering criteria based on expression thresholds and on the recurrence of the candidate junction-peptide across the cancer and normal cohorts (Fig. 1h). Tumor-associated *k*-mers can be obtained by removing *k*-mers present in the desired background cohort in *u* samples at any read level (recurrence parameter) or expressed with at least *R* reads. *ImmunoPepper* additionally allows the filtering of the candidate peptides based on their sequence occurrence in a user-specified background set (Supplementary Methods, available as supplementary data at *Bioinformatics* online).

Cancer support-level can be set by only including *k*-mers with an expression higher or equal than the threshold *T* in at least *l* samples (recurrence parameter) excluding a “target” sample. The expression threshold for the “target” sample can be set with the read threshold *t* (Supplementary Methods, available as supplementary data at *Bioinformatics* online).

Expression normalization can be applied prior to applying the thresholds. For the recurrence parameters, a whitelist for the set of samples to include can be provided as a parameter. More advanced filtering options are available based on the presence of the reading frame or of the junctions in the annotation file.

#### 3.1.3 Filtering of peptides based on MHC-binding

To assess whether a peptide can be presented to T-cells, which is required for a neoepitope, *ImmunoPepper* allows the user to perform MHC-I and MHC-II binding prediction (Fig. 1i) with a large set of MHC-binding prediction tools: *MHCflurry* (O’Donnell *et al.* 2020), *NetMHC3* (Lundegaard *et al.* 2008), *NetMHC4* (Andreatta and Nielsen 2016), *NetMHCpan*, *NetMHCIIpan* (Jurtz *et al.* 2017), *NetMHCcons* (Karosiene *et al.* 2012), *IedbMhcClass1* and *IedbMhcClass2*  http://tools.iedb.org/mhci/, via the mhctools interface. A comparison of MHC-binding software tools implemented by several pipelines can be found in Table 4, available as supplementary data at *Bioinformatics* online.

Our wrapper adds two features useful in the context of neoepitope candidate prediction: (i) A possibility to pass relevant metadata columns regarding the candidate characteristics (e.g. expression, recurrence, coordinates) to be displayed next to the binding prediction results of the software. Those are output in a table together with the MHC-binding scores. (ii) An option to apply thresholds on the MHC-binding scores.

#### 3.1.4 Validation of peptides with mass spectrometry

Finally, *ImmunoPepper* enables the user to search for protein expression of the candidates in mass spectrometry data (Fig. 1j). Currently, only the IRIS (Pan *et al.* 2023) software tool offers some integrated MS validation. *ImmunoPepper* provides a wrapper to *PepQuery* (Wen *et al.* 2019), a peptide-centric mass spectrometry software. *PepQuery* matches experimental spectra to a predicted spectrum for the target peptide, and compares it to a match between the experimental spectra and a predicted spectrum for each competition peptide. Three successive competition steps (Wen *et al.* 2019) are available. The output from our wrapper is a user-readable summary file indicating whether the target peptide has a mass spectrometry match and which of the competition steps are satisfied. We also publish the scripts for running the *Subset-Neighbor-Search* (Lin *et al.* 2021), a target-decoy competition (TDC) algorithm suitable for the discovery of rare peptides, and plan their direct integration into *ImmunoPepper*.

### 3.2 Software availability

*ImmunoPepper* is available via the Python package index (PyPI) and its source code is freely available under an open-source license. The documentation of input and output parameters as well as tutorials are available online at https://immunopepper.readthedocs.io/en/latest/.

## 4 Experiments

### 4.1 Experiment setup

#### 4.1.1 ImmunoPepper inputs

Germline and somatic variant calling for 32 ovarian cancer (TCGA-OV) and 31 breast invasive carcinoma (TCGA-BRCA) was performed as in Kahles *et al.* (2018). TCGA cancer samples and GTEx normal samples were processed with *SplAdder* (Kahles *et al.* 2016) in the same way as in Kahles *et al.* (2018). Besides, the TCGA graph was also re-processed with *SplAdder* to quantify each of the junctions from the cancer graph based on the GTEx graph expression values.

#### 4.1.2 *Foreground* cohort filtering and *background* filtering

The same *foreground* cohort criteria of Kahles *et al.* (2018) were used. The analysis focuses on *junction 9-mers* for consistency with Kahles *et al.* (2018), but 8-mers or 10-mers are also biologically-relevant choices supported by *ImmunoPepper*. The *junction 9-mers* from the *foreground* cohort are included if expressed with any read count in the *foreground* sample or if the maximal expression across the cohort was at least 20 normalized reads. The normalization was performed by multiplying the same sample normalization factor values as in Kahles *et al.* (2018). The factors correspond to the library sizes per sample divided by the median library size across the *foreground* cohort. After application of *foreground* cohort filtering, the *background* of normal peptides was removed. Two different experimental setups named *re-quant* and *all-frame* pipelines were implemented to showcase the dimensions of variation of our algorithm. Motivation for the methods is described in Section 4.2 and details of implementation can be found in the Supplementary Methods, available as supplementary data at *Bioinformatics* online.

#### 4.1.3 MHC-binding analysis

The patient-specific MHC alleles were obtained as in Kahles *et al.* (2018). Then, 9-mers passing the filtering criteria on the *foreground* cohort and on the *background* GTEx cohort (strict exclusion of normal 9-mers) were tested for MHC-I binding with the *NetMHC4* prediction tool via the *ImmunoPepper* wrapper. 9-mers with binding affinity scores in the top 2% (*binding rank*) were considered.

#### 4.1.4 Mass spectrometry analysis

Mass spectrometry confirmation of candidate *junction 9-mers* was assessed by generating trypticjunction-peptides associated with the candidate 9-mers, and testing the presence of the tryptic junction-peptides in the Clinical Proteomic Tumor Analysis Consortium (CPTAC) (Rudnick *et al.* 2016) mass spectrometry dataset (MS) from the corresponding patients. First, the junction 9-mers were mapped to their context 2-exon (or 3-exon) peptides. Then the peptides were *in silico* digested with trypsin. Finally, the tryptic peptides overlapping the junctions’ genomic positions were extracted. The tryptic-peptides were defined as the peptides resulting from two tryptic cleavages, or the peptides from the start of the transcript to the first tryptic-cleavage site or from the last tryptic-cleavage site to the end of the transcript. The tryptic junction-peptides’ presence in the MS data was searched both with the *PepQuery* (Wen *et al.* 2019) engine and the *Subset-Neighbor-Search* (Lin *et al.* 2021) approach (Supplementary Methods, available as supplementary data at *Bioinformatics* online).

### 4.2 Extraction of personalized peptides in the *re-quant* and *all-frames* experimental setups

We generated peptide lists for 32 OV and 31 BRCA samples as well as for 3233 GTEx samples (Lonsdale *et al.* 2013) in a similar setup as in previously published analyses (Kahles *et al.* 2018).

To better understand the behavior of our algorithm with regard to generation of candidates from novel splice junctions and novel reading frames, we propose the *re-quant* and the *all-frames* experiments. Briefly, in the *re-quant* experiment: *Foreground* 9-mers are filtered out by quantifying if the same junction position in the *background* GTEx graph has any overlapping reads. In the *all-frames* experiment: *Foreground* 9-mers are filtered out sequence-wise against 9-mers from the *background* GTEx normal cohort translated using all possible reading frames. With the *all-frames* method, we can quantify the portion of candidates arising strictly from novel cancer junctions. With the *re-quant* experimental setup, we can quantify the portion of candidates arising from novel junctions and from known junctions with a novel reading frame. Importantly, the germline variants also supported by the GTEx cohort are excluded (Supplementary Methods, available as supplementary data at *Bioinformatics* online for *all-frames* and *re-quant*). This step ensures that these 9-mers are not reported as tumor-specific candidates in the output.

**Figure 2.**
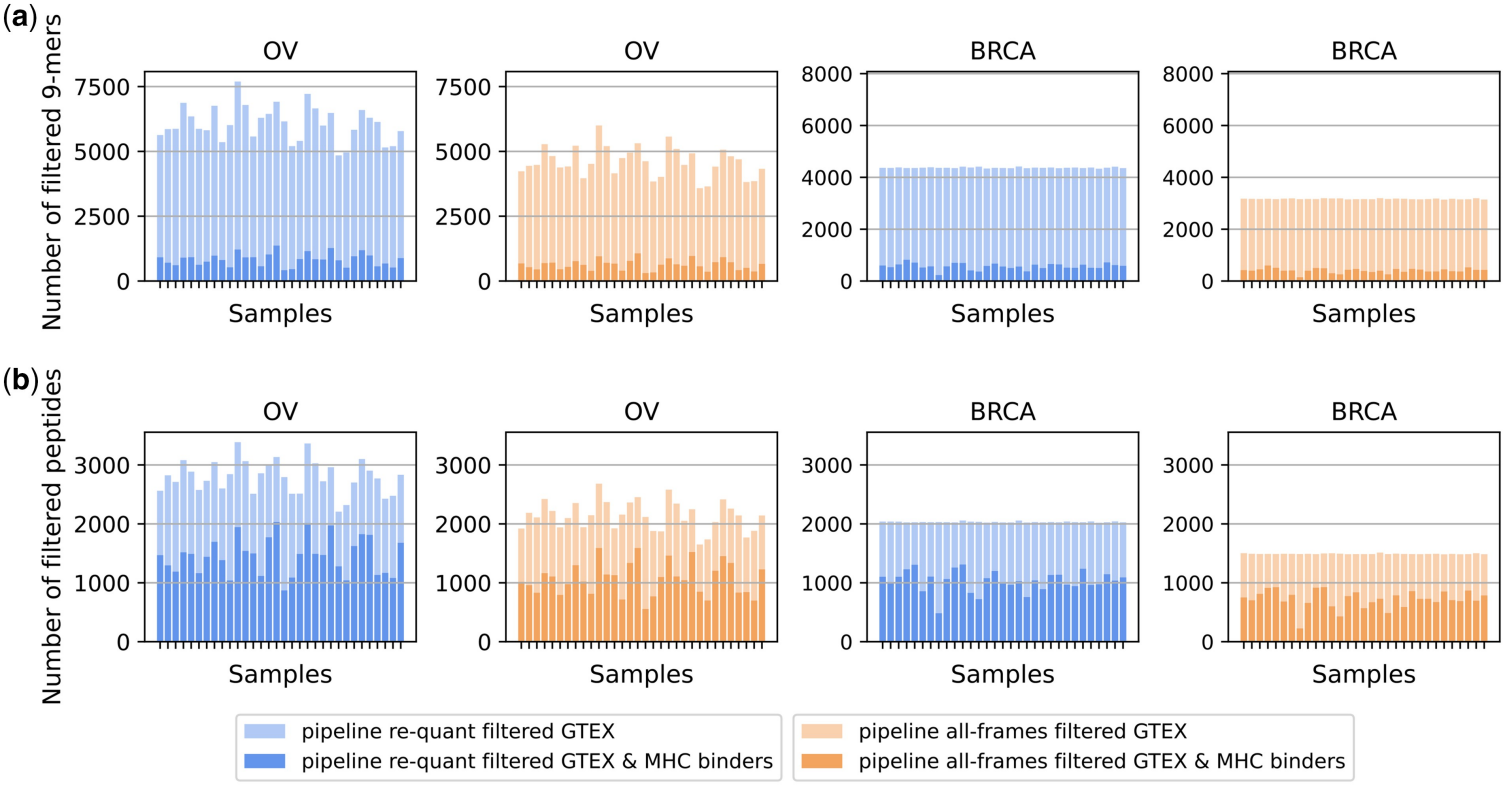
Number of 9-mers and 2-exon peptide candidates generated from the cancer samples after removing 9-mers present in GTEx and applying cancer support filtering criteria. (a) Number of 9-mers. (b) Number of 2- or 3-exons peptides. Blue (columns 1 and 3): Output of pipeline *re-quant*. Orange (columns 2 and 4): Output of pipeline *all-frames*. Lighter alpha: no restriction on MHC-binding. Darker alpha: restriction on MHC-binding.

After filtering, we found more than 3582 9-mers with the *all-frames* setup and more than 4847 9-mers with the *re-quant* setup (Fig. 2a). When restricting the filtered set for MHC-binding properties, the number of 9-mers was between 307 and 1067 for the *all-frames* method and between 419 and 1367 for the *re-quant* setup, respectively (Fig. 2a). Similar candidate numbers between the BRCA samples are due to the filtering criteria implemented (Supplementary Methods, available as supplementary data at *Bioinformatics* online). The number of translated 2-exon (and 3-exons) peptides, or “context” peptides (Section 3.1.1) for the 9-mers, is reported in Fig. 2b. Besides, we compared *re-quant* and *all-frames* for 9-mer identity and observed that the candidates from *all-frames* are included in the set of candidates for *re-quant* (Fig. 1, available as supplementary data at *Bioinformatics* online). This demonstrates that our *re-quant* approach includes additional variability from the propagated frames, as expected.

To display *ImmunoPepper’*s functionality of integrating personalized mutations and alternative splicing, we analyzed the type of candidates passing the filtering criteria (*re-quant* experiment). In the OV cohort, 73 153 germline variants per sample (median), and 78 and 229 somatic mutations in two samples were input to *ImmunoPepper*. In the BRCA cohort, 92 158 germline variants per sample (median) and 195 and 92 somatic variants in two distinct samples were used. We observed a median of 67 and 56 filtered 9-mer containing germline variants per sample for the OV and the BRCA cohort, respectively (Fig. 7a, available as supplementary data at *Bioinformatics* online). In each sample, 9 and 8 candidate 9-mers (median) had MHC-binding properties in the OV and BRCA cohorts, respectively (Fig. 7b, available as supplementary data at *Bioinformatics* online). In the OV and BRCA samples, we found a median of 7 filtered 9-mers containing somatic mutations per sample (Fig. 7a, available as supplementary data at *Bioinformatics* online). When restricting for MHC-binding, this number dropped to 1 per sample (Fig. 7b, available as supplementary data at *Bioinformatics* online). No tumor-associated 9-mer containing both types of variants was found, likely due to the reduced number of somatic mutations. In contrast, we found 5934.5 (823.69 MHC-binders) and 4303 (558 MHC-binders) filtered splicing-only-derived 9-mers per sample (median) in OV and BRCA samples, respectively (Fig. 7a and b, available as supplementary data at *Bioinformatics* online). For the analysis of non-junction candidates with germline or somatic variation only, we refer to Kahles *et al.* (2018). Pure germline variants are usually not of interest for neoepitope candidate generation, as they are not cancer-cell specific.

### 4.3 Peptides extracted with ImmunoPepper reach better validation with proteomics data

MS detects potential expression of *in-silico*-derived candidates at the peptide level. This orthogonal observation of the candidates allows for increased confidence in the predictions. MS data analysis relies on assignment of a peptide sequence to an experimental spectrum; a theoretical spectrum is derived from the peptide candidate and its similarity to an experimental spectrum is computed. In the case of rare novel target peptides for immunotherapy, the difficulty of the detection lies in (i) the disentanglement between normal and rare peptides, as wrong assignments of rare target peptides to normal instances commonly occur, and (ii) in achieving sufficient discoveries while keeping the false positive rate low. To better understand these steps, we compared the discoveries made with a peptide-centric tool called *PepQuery* (Wen *et al.* 2019) and a target-decoy competition (TDC) search framework named *Subset-Neighbor-Search* (*SNS*) (Lin *et al.* 2021). *PepQuery* includes one competition step against normal peptides and one competition step against normal peptides bearing post-translational modifications. *SNS* restricts the peptide database to rare target peptides and their neighbors derived from the normal proteome which acts as an implicit normal competition step.

**Figure 3.**
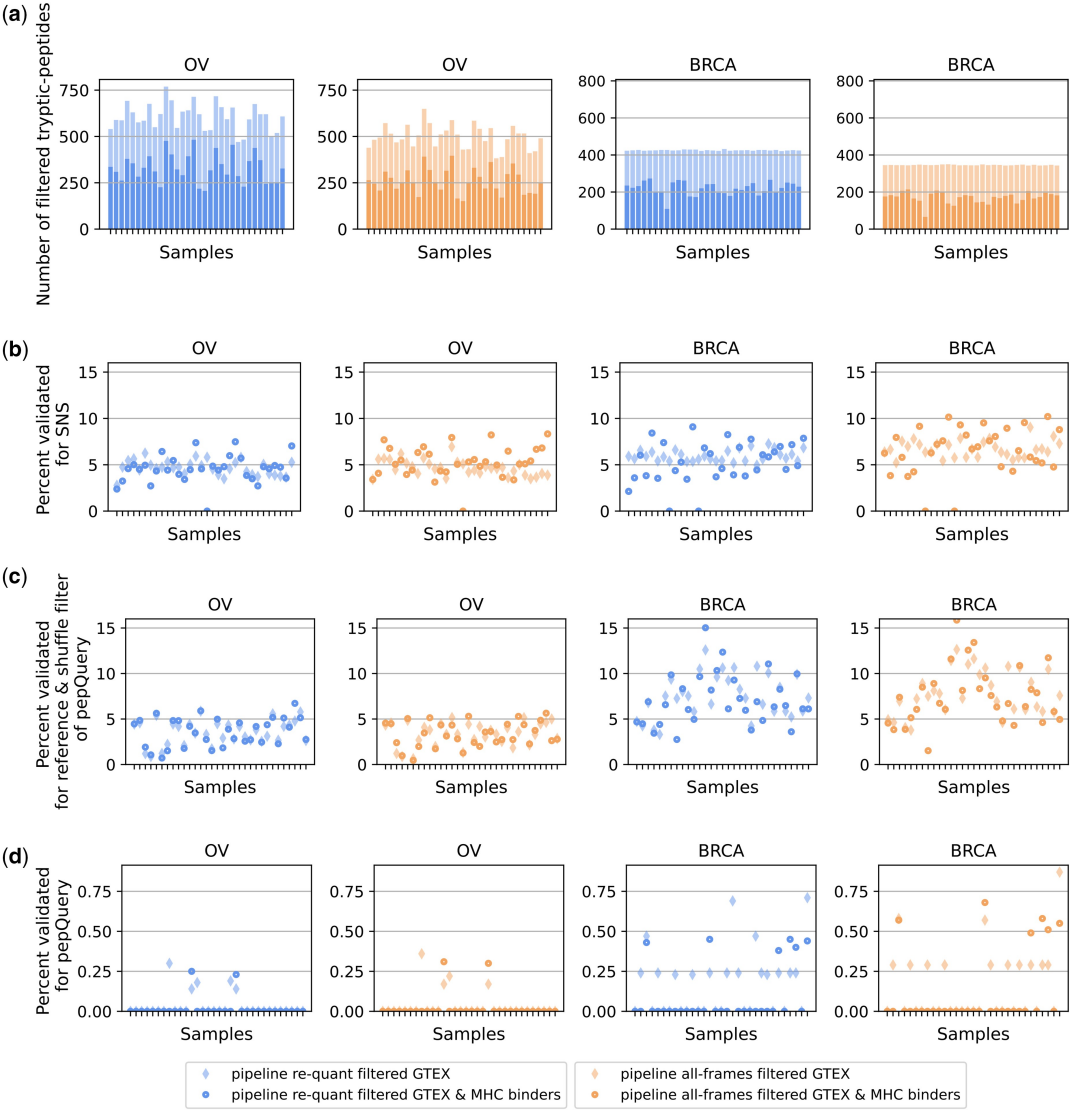
Peptide candidates confirmed with mass spectrometry (MS). (a) Number of filtered tryptic junction-peptides provided as input to MS analyses. (b) Percent of tryptic junction-peptides validated with the *Subset-Neighbor-Search* (*SNS*) method. (c) Percent of tryptic junction-peptides validated with the *PepQuery* method without post-translational modification competition step. (d) Percent of tryptic junction-peptides validated with the *PepQuery* method with post-translational modification competition step. Blue (columns 1 and 3): Output of pipeline *re-quant*. Orange (columns 2 and 4): Output of pipeline *all-frames*. Lighter alpha: no restriction on MHC-binding. Darker alpha: restriction on MHC-binding.

Both MS analysis algorithms were fed tryptic junction-peptides as input. The input numbers varied from 382 to 769 per sample across the OV and BRCA cohorts (across *re-quant* and *all-frames* setups). Restricting the input to MHC-binders only, 151 to 482 tryptic junction-peptides were present in the OV and BRCA cohorts, respectively (Fig. 3a).

The percentage of tryptic junction-peptides confirmed with MS (resp. MHC-binding tryptic junction-peptides) varied from 2.8% to 9% (resp. 0% to 10.2%) per sample for the *SNS* method (Fig. 3b). We looked at the proportion of confirmed peptides when running *PepQuery* without the post-translational modification competition step because *PepQuery* does not apply False Discovery Rate (FDR) estimation, which is especially problematic when post-translation-modified by-products of major proteins could compete with neoantigens (Methods 2.5, available as supplementary data at *Bioinformatics* online). In this setup, we found a validation rate for tryptic junction-peptides (resp. MHC-binding tryptic junction-peptides) between 0.6% and 12.6% per sample (resp. 0.5% to 15.9%) (Fig. 3c). These numbers were overall higher than the “expected” validations rates of 0% to 3% that we estimated from the literature (Supplementary Methods, available as supplementary data at *Bioinformatics* online).

From the validated tryptic junction-peptides, we extracted the tumor-associated validated 9-mers candidates for the *re-quant* and *all-frames* pipelines. For the *SNS* method, 80 to 236 candidates per sample were found across both cohorts (0 to 54 were MHC-binders) across both pipelines (Fig. 2a, available as supplementary data at *Bioinformatics* online), of which, at most six candidates per sample were 9-mers containing a splicing-junction and germline variant(s) (non-MHC-binding 9-mers). The remaining candidates were found to be splicing-derived only (Fig. 8a and b, available as supplementary data at *Bioinformatics* online). For *PepQuery* without the post-translational modification competition step 15 to 336 9-mers per sample were validated (1–52 MHC-binders) (Fig. 2c, available as supplementary data at *Bioinformatics* online), with at most six candidates per sample derived from splicing and germline variation, among which two candidates with MHC-binding properties. The rest were splicing-derived (Fig. 9a and b, available as supplementary data at *Bioinformatics* online).

We assessed the set intersection between the different proteomics methods. In the OV cohort, 5/24 samples shared candidates across the two methods, corresponding to 32 shared 9-mer candidates across all samples. This represents only a small portion of the total validated candidates across samples: 735 for the *SNS* and 1542 for the *PepQuery* without the post-translational modification step. In the BRCA cohort, 10/19 samples shared candidates across the two methods, corresponding to 56 shared 9-mer candidates across all samples for a total candidate number across samples of 374 for the *SNS* and 1126 for the *PepQuery* without the post-translational modification step. When applying the MHC-binding criteria on the input of the proteomics search, seven shared 9-mer candidates across all OV samples were found (total: 207 for the *SNS*, 337 for the *PepQuery* without the post-translational modification step) and eight shared 9-mer candidates across all BRCA samples were found (total: 96 for the *SNS* and 234 for the *PepQuery* without the post-translational modification step).

Finally, we designed a negative control experiment that tests MS support of peptides translated exclusively in reading frames which are not present in the annotation file (Methods 2.5.3, available as supplementary data at *Bioinformatics* online). We report numbers for the *re-quant* pipeline (for both pipelines see Fig. 5, available as supplementary data at *Bioinformatics* online), and compare it to the positive experiment (P.E.) (Fig. 3). With the SNS method, the percentage of tryptic junction-peptides confirmed with MS was on average 0.01 (4.66 for the P.E.) for the OV and 0.01 (5.94 P.E.) in the BRCA cohort. On average, 0 (4.52 P.E.) and 0.01 (5.26 P.E.) MHC-binding tryptic junction-peptides were confirmed in the OV and BRCA cohorts. With the *PepQuery* method without the post-translational modification step, the percentage of tryptic junction-peptides confirmed with MS was on average 1.88 (3.54 for the P.E.) for the OV and 3.85 (7.55 P.E.) in the BRCA cohort. On average, 3.5 (3.6 P.E.) and 7.65 (7.1 P.E.) MHC-binding tryptic junction-peptides were confirmed in each of the cohorts; in this case, the maximum values of the P.E. were above the values of the negative control experiment by about 10%. The trends for the *PepQuery* method with the post-translational modification step were similar.

### 4.4 ImmunoPepper closely reproduces previously published results

In the following experiments, we aim to understand how the new software compares with the previously published pipeline (Kahles *et al.* 2018). On the methodological side, the published analysis (Kahles *et al.* 2018) builds splicing graphs of the normal and the cancer cohort separately followed by MS analysis and filtering. Here, we perform the filtering prior to the MS analysis. On the normal side, we used the same splicing graph as previously published, and on the cancer side, we used an equivalent graph. The peptide translation is now performed with the *ImmunoPepper* tool which provides a richer and more sensitive set of candidates. The filtering in our analysis is performed following the *re-quant* and the *all-frames* heuristics, which are slightly more stringent versions of the filtering in Kahles *et al.* (2018).

**Figure 4.**
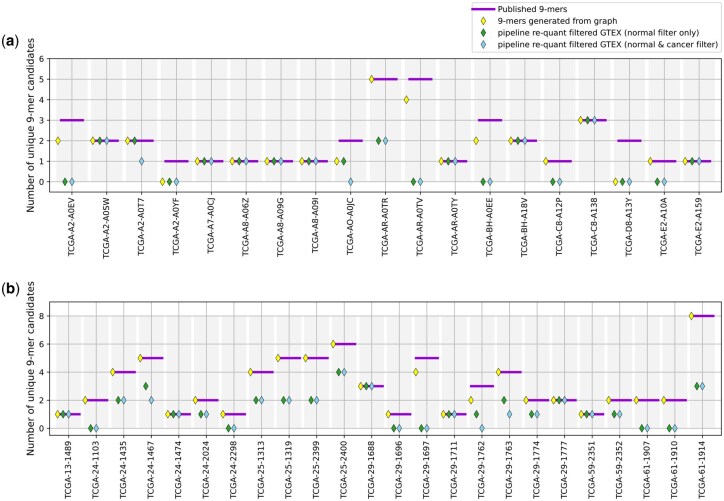
Recovery of previously published candidate peptides (Kahles *et al.* 2018). The 9-mers are generated from the *merged* splicing graph, then 9-mers present in the GTEx cohort are filtered out. Finally, expression and recurrence criteria are applied in the cancer cohorts. Recovery in BRCA (a) and OV (b) samples.

We first assessed the recovery of the previously published (Kahles *et al.* 2018) neoantigens after the generation and the filtering steps of our analysis (Fig. 4 and Fig. 6, available as supplementary data at *Bioinformatics* online). Exact recovery is the recovery of all neoantigens of the previous publication, while partial recovery refers to a non-empty intersection between the predictions. We observed that after generating a splicing graph with the TCGA cancer sample and translating peptides with *ImmunoPepper* 22/24 and 13/19 samples for the OV and the BRCA cohort, respectively, had exact recovery of the published candidates, and 24/24 and 17/19 had at least partial recovery in OV and BRCA cohorts, respectively (Fig. 4). After applying the GTEx filter either in the *re-quant* or the *all-frames* setup 18/24 samples for the OV cohort and 12/19 for the BRCA cohort had at least partial recovery. Finally, this ratio goes to 17/24 samples for the OV cohort and 11/19 samples for the OV cohort after applying the cancer filtering/cancer recurrence criteria on top of the GTEx filter (Fig. 4, Fig. 6, available as supplementary data at *Bioinformatics* online).

Next, we assessed the recovery after the proteomics step. In the previously published results (Kahles *et al.* 2018), the MS analysis was performed with the MS-GF+ (Kim and Pevzner 2014) suite. It is now performed with the *SNS* and *PepQuery* methods. MS-GF+ (Kim and Pevzner 2014), a target-decoy competition approach, is closest to the *SNS* framework. We expect *SNS* to be more sensitive than MS-GF+, although we have observed that (i) MS search techniques do not seem to be consistent in peptide discoveries and (ii) performing the MS analysis with a larger input set (for example earlier in the pipeline), will lead to a higher absolute number of confirmed peptides.

After application of the *SNS* step in the pipeline, we observed a partial recovery of the previously published candidates in 2/24 OV samples and 3/19 samples in the BRCA cohorts. For the OV cohort, this corresponds to the candidates “YEGYSFTTT,” “YKSLQVAEV,” and for the BRCA cohort, to the candidates “QTVDLFEGR,” “VLCEVFKYI,” “IYEGYSFTT.” Restricting the input to MHC-binders only led to recovery in 1/24 OV samples and 2/19 samples BRCA samples, respectively. When the *PepQuery* method without post-translational modifications was applied, recovery of “YKSLQVAEV” was observed in 1/24 OV samples and 0/19 BRCA samples, respectively. This result did not change after applying the MHC-binding criteria.

## 5 Discussion

We presented *ImmunoPepper*, a novel software tool for the prediction of personalized, putative neoepitopes, which not only takes genomic variability but also alternative splicing and alternative transcript processing into account. As output, it provides the user with a set of candidate neoepitopes and metadata including the expression level in the sample and its recurrence in the cancer cohort. Moreover, the presence of the candidate’s exon–exon junction and reading frame in canonical transcripts of the annotation file is reported. Additional metrics related to immunogenicity include the candidate’s MHC-binding rank and its IC50. Finally, *ImmunoPepper* allows the integration of proteomics data for assessment of the protein-level presence of the candidate.

*ImmunoPepper* has several unique features compared to previous works. First, it performs inclusion of splicing variants jointly with germline individual variation and somatic mutations from user-provided variant calls. Besides, it includes a novel graph translation algorithm for generation of candidates. To date, only Neosplice (Chai *et al.* 2022) and moPepGen (Zhu *et al.* 2024), a parallel work, currently without peer review, allow some form of reading frame propagation along graph paths. This generation algorithm models the splicing process and translates the peptides across all possible reading frames or follows “annotated” reading frames from an external annotation file. Translation following annotated reading frames (Zhang *et al.* 2020, Chai *et al.* 2022, Pan *et al.* 2023), and all possible reading frames translation (Li *et al.* 2024) have been proposed, but no tool outside *ImmunoPepper* and moPepGen (Zhu *et al.* 2024) currently supports both. Additionally, *ImmunoPepper* offers several filtering options for the candidates. Interestingly, the works describing the software tools SNAF (Li *et al.* 2024), IRIS (Pan *et al.* 2023), Neosplice (Chai *et al.* 2022), and ASNEO (Zhang *et al.* 2020) chose to allow a tolerance of 1–4 reads in the normal background cohort. We think that allowing the user to set lenient or strict filtering criteria is important to control the risk of off-target effects of cancer vaccines in normal tissues. Hence, we decided for a complete exclusion of any candidate found in the background normal cohort in our results. Finally, *ImmunoPepper* is particularly well suited for proteogenomics analyses. Its longer translation context allows both the *k*-merization of the peptides as well as the *in silico* enzymatic digestion of the output prior to MS validation.

To better understand the output space of candidates, we performed experiments that segregate different variability dimensions generated by the software tool. The *re-quant* experimental setup includes only candidates arising from novel junctions and from known junctions with a novel reading frame, whereas the *all-frames* setup, includes only candidates arising strictly from novel cancer junctions. In this way, the *re-quant* approach quantifies a cancer foreground splice graph in a cohort of normal samples, while the *all-frames* approach separately translates the cancer and normal candidates. The *re-quant* approach is more sensitive, and allows strong specificity with limited computation. Moreover it is faster to run, as it does not require translation of the complete GTEx graph. In general, we recommend generating cancer and normal epitopes via a common data structure (e.g. a merged splicing graph for cancer and normal samples, or a re-quantified cancer splicing graph) to achieve maximal sensitivity while controlling specificity. Indeed, in the case where the processing criteria are stricter on the normal set than on the cancer set, false positive tumor-associated neoepitope candidates can be generated. A potential limitation is the dependency of the sensitivity and specificity on *SplAdder* (Kahles *et al.* 2016). The user can decide via *SplAdder* on an appropriate level of sensitivity.

We analyzed OV and BRCA TCGA tumor samples with a strict tumor-associated filtering configuration and found RNA-seq-supported and tumor-associated candidates, among which a set of candidates also satisfying the binding to the MHC-I complex binders. Then, we MS validated the candidate’s presence with a peptide-centric and a target-decoy competition-based method: the *PepQuery* and the *SNS*.

In the MS validation, we observed validation rates that were above the expected validation rates of 0% to 3% for proteogenomics analyses (Supplementary Results, available as supplementary data at *Bioinformatics* online), and way above the splicing-derived neoepitope candidate validation percent estimated from Ruggles *et al.* (2016) and Pan *et al.* (2023) of up to 0.29%. Indeed, the percentage of MHC-binding tryptic junction-peptides confirmed with MS was as high as 10.2% per sample for the *Subset-Neighbor-Search* (*SNS*) method and 15.9% for *PepQuery*. The two methods showed overall comparable validation rates, but limited overlap of validated candidates. The discrepancy could be due to the different assumptions behind peptide-centric and target-decoy competition frameworks. Nevertheless, the fact that *PepQuery* validates significantly more negative control peptides than *SNS*, suggests that the target-decoy experiments have a better specificity. This result highlights the importance of understanding the assumptions made by MS analysis algorithms and the need for false discovery rate control in the validation experiments. No study so far included both TDC and peptic-centric proteomics analyses for splicing-derived neoepitope candidate validation. A study that included more than one proteomics validation approach but did not compare them presented the ScanNeo (Wang *et al.* 2019) algorithm for INDEL-derived neoepitope candidate generation.

We found that very few published candidates found with MS (Kahles *et al.* 2018) could be validated. Given that the mass spectrometry analysis was performed prior to filtering (Kahles *et al.* 2018), this suggests that application of the proteomics step seems to be very sensitive to its placement in the stage of the pipeline. Therefore, we caution against comparing proteomics validation rates between pipelines whose validation algorithms or order of validation steps greatly differ.

Notably, proteomics analyses presented in previously published pipelines lack consistency, creating additional variability. For instance, several studies applied a FDR threshold of 5% (Pan *et al.* 2023, Li *et al.* 2024) while others applied a threshold of 1% (Zhang *et al.* 2020, Zhu *et al.* 2024) or, in some cases, did not perform a FDR correction at all (Chai *et al.* 2022). Moreover, the search database often varied greatly, with the inclusion of normal peptides and predicted neoepitope candidates (Zhang *et al.* 2020, Li *et al.* 2024), or the sole inclusion of neoepitope candidates (Li *et al.* 2024). Notably, the latter database does not control properly for the FDR (Lin *et al.* 2021). Other studies (Pan *et al.* 2023, Li *et al.* 2024) have used immunopeptidomics data as the validation layer. This choice, when available, is good, although it is more biased toward abundant peptides (Bassani-Sternberg 2018). Overall, MS validation of neoepitope candidates is a difficult problem because neoepitopes are rare across cohorts and can be lowly expressed and estimating the “true” validation rate from such diverse protocols is non-trivial.

Notably, previously published pipelines did not assess reproducibility. In our work, we have shown how *ImmunoPepper* carefully builds on previous results (Kahles *et al.* 2018) and suggested that the direct comparison of splicing-derived neoepitope pipelines is a difficult task. We do not propose a full benchmark analysis in this work because it would require a thorough assessment of meaningful parameters to compare between various tools (Prélot and David 2024). To maximize reproducibility of neoepitope analyses, we advise users to (i) use the same input samples for cancer and normal samples, (ii) to align the order of pipeline steps, (iii) to use the same software version for similar steps, such as the alignment, the variant calling or the MHC-binding steps, and (iv) use similar levels of stringency for the filtering steps. We should note that, as the reproducibility of results published in earlier studies was an important focus of our analysis, we did not filter against a larger GTEx cohort. This is a less stringent filter than possible, but do not expect dramatically different results when using a larger GTEx background. In this case, one would expect a drop in the number of candidates, validation of some candidates will still be possible. This analysis was performed by (Prélot and David 2024) with almost 11 000 samples in the GTEx background. The computational features of the *ImmunoPepper* software tool that allow running such a large analysis are discussed in the Supplementary Methods, available as supplementary data at *Bioinformatics* online.

In the future, *ImmunoPepper* could be leveraged for the design of personalized cancer vaccines by integration in a container application for portable reproducible results (Richters *et al.* 2019). Further features could include the integration of immunogenicity prediction (Richman *et al.* 2019) or the use of long-read sequencing data (Au *et al.* 2013, Sharon *et al.* 2013). In future work, the effect of different filtering parameters on the overall results should also be carefully assessed. Experimental validation with targeted proteomics and spike-in peptides could help validate the presence of the candidates in the data, while T-cell reactivity assays could further provide a “ground truth” for candidates. However, these techniques remains low-throughput and take place in an external system instead of the patient of interest. Therefore, we advocate for the use of multiple-software tools for the generation of candidate neoepitopes, similar to the strategy used in the variant calling field (Koboldt 2020).

## Supplementary Material

btaf492_Supplementary_Data

## Data Availability

The data is available via the database of Genotypes and Phenotypes (dbGaP) http://www.ncbi.nlm.nih.gov/gap with accession numbers phs000424.v9.p2 and phs000178.v11.p8 for GTEx and TCGA, respectively. Information about GTEx can be found at http://commonfund.nih.gov/GTEx and information about TCGA can be found at http://cancergenome.nih.gov. The list of donor ID and cancer type of samples used for CPTAC-based peptide verification can be found at https://api.gdc.cancer.gov/data/5c9ec65d-57c8-44f8-b71e-e612a6e6f0a6, and the list of CPTAC datasets used per donor can be found at https://api.gdc.cancer.gov/data/6eca6de5-9be0-4a3c-93e6-012752cf21c0.

## References

[btaf492-B1] Andreatta M , Nielsen M. Gapped sequence alignment using artificial neural networks: application to the MHC class I system. Bioinformatics 2016;32:511–7. 26515819 10.1093/bioinformatics/btv639 PMC6402319

[btaf492-B2] Au KF , Sebastiano V, Afshar PT et al Characterization of the human ESC transcriptome by hybrid sequencing. Proc Natl Acad Sci USA 2013;110:E4821–30. 24282307 10.1073/pnas.1320101110 PMC3864310

[btaf492-B3] Bassani-Sternberg M. Mass spectrometry based immunopeptidomics for the discovery of cancer neoantigens. Methods Mol Biol 2018;1719:209–21. 29476514 10.1007/978-1-4939-7537-2_14

[btaf492-B4] Bjerregaard A-M , Nielsen M, Hadrup SR et al MuPeXI: prediction of neo-epitopes from tumor sequencing data. Cancer Immunol Immunother 2017;66:1123–30. 28429069 10.1007/s00262-017-2001-3 PMC11028452

[btaf492-B5] Chai S , Smith CC, Kochar TK et al NeoSplice: a bioinformatics method for prediction of splice variant neoantigens. Bioinform Adv 2022;2:vbac032. 35669345 10.1093/bioadv/vbac032 PMC9154024

[btaf492-B6] Chong C , Müller M, Pak H et al Integrated proteogenomic deep sequencing and analytics accurately identify non-canonical peptides in tumor immunopeptidomes. Nat Commun 2020;11:1293. 32157095 10.1038/s41467-020-14968-9 PMC7064602

[btaf492-B7] Cotto KC , Feng Y-Y, Ramu A et al Integrated analysis of genomic and transcriptomic data for the discovery of splice-associated variants in cancer. Nat Commun 2023;14:1589. 36949070 10.1038/s41467-023-37266-6 PMC10033906

[btaf492-B8] Guo C , Manjili MH, Subjeck JR et al Therapeutic cancer vaccines: past, present, and future. Adv Cancer Res 2013;119:421–75. 23870514 10.1016/B978-0-12-407190-2.00007-1 PMC3721379

[btaf492-B9] Hu A , Noble WS, Wolf-Yadlin A. Technical advances in proteomics: new developments in data-independent acquisition. F1000Res 2016;5. 10.12688/f1000research.7042.1 PMC4821292 27092249

[btaf492-B10] Hundal J , Kiwala S, McMichael J et al pVACtools: a computational toolkit to identify and visualize cancer neoantigens. Cancer Immunol Res 2020;8:409–20. 31907209 10.1158/2326-6066.CIR-19-0401 PMC7056579

[btaf492-B11] Jurtz V , Paul S, Andreatta M et al NetMHCpan-4.0: improved peptide-MHC class I interaction predictions integrating eluted ligand and peptide binding affinity data. J Immunol 2017;199:3360–8. 28978689 10.4049/jimmunol.1700893 PMC5679736

[btaf492-B12] Kahles A , Ong CS, Zhong Y et al SplAdder: identification, quantification and testing of alternative splicing events from RNA-Seq data. Bioinformatics 2016;32:1840–7. 26873928 10.1093/bioinformatics/btw076 PMC4908322

[btaf492-B13] Kahles A , Lehmann K-V, Toussaint NC, et al; Cancer Genome Atlas Research Network. Comprehensive analysis of alternative splicing across tumors from 8,705 patients. Cancer Cell 2018;34:211–24.e6. 30078747 10.1016/j.ccell.2018.07.001 PMC9844097

[btaf492-B14] Karosiene E , Lundegaard C, Lund O et al NetMHCcons: a consensus method for the major histocompatibility complex class I predictions. Immunogenetics 2012;64:177–86. 22009319 10.1007/s00251-011-0579-8

[btaf492-B15] Kim S , Pevzner PA. MS-GF+ makes progress towards a universal database search tool for proteomics. Nat Commun 2014;5:5277. 25358478 10.1038/ncomms6277 PMC5036525

[btaf492-B16] Kim S , Kim HS, Kim E et al Neopepsee: accurate genome-level prediction of neoantigens by harnessing sequence and amino acid immunogenicity information. Ann Oncol 2018;29:1030–6. 29360924 10.1093/annonc/mdy022

[btaf492-B17] Koboldt DC. Best practices for variant calling in clinical sequencing. Genome Med 2020;12:91. 33106175 10.1186/s13073-020-00791-w PMC7586657

[btaf492-B18] Lang F , Sorn P, Suchan M et al Prediction of tumor-specific splicing from somatic mutations as a source of neoantigen candidates. Bioinform Adv 2024;4:vbae080. 38863673 10.1093/bioadv/vbae080 PMC11165244

[btaf492-B19] Laumont CM , Vincent K, Hesnard L et al Noncoding regions are the main source of targetable tumor-specific antigens. Sci Transl Med 2018;10:eaau5516. 30518613 10.1126/scitranslmed.aau5516

[btaf492-B20] Li G , Mahajan S, Ma S et al Splicing neoantigen discovery with SNAF reveals shared targets for cancer immunotherapy. Sci Transl Med 2024;16:eade2886. 38232136 10.1126/scitranslmed.ade2886 PMC11517820

[btaf492-B21] Lin A , Plubell DL, Keich U et al Accurately assigning peptides to spectra when only a subset of peptides are relevant. J Proteome Res 2021;20:4153–64. 34236864 10.1021/acs.jproteome.1c00483 PMC8489664

[btaf492-B22] Lonsdale J , Thomas J, Salvatore M et al The genotype-tissue expression (gtex) project. Nat Genet 2013;45:580–5. 23715323 10.1038/ng.2653 PMC4010069

[btaf492-B23] Lundegaard C , Lamberth K, Harndahl M et al NetMHC-3.0: accurate web accessible predictions of human, mouse and monkey MHC class I affinities for peptides of length 8-11. Nucleic Acids Res 2008;36:W509–12. 18463140 10.1093/nar/gkn202 PMC2447772

[btaf492-B24] O’Donnell TJ , Rubinsteyn A, Laserson U. MHCflurry 2.0: improved pan-allele prediction of MHC class I-presented peptides by incorporating antigen processing. Cell Syst 2020;11:42–8.e7. 32711842 10.1016/j.cels.2020.06.010

[btaf492-B25] Pan Y , Phillips JW, Zhang BD et al IRIS: discovery of cancer immunotherapy targets arising from pre-mRNA alternative splicing. Proc Natl Acad Sci USA 2023;120:e2221116120. 37192158 10.1073/pnas.2221116120 PMC10214192

[btaf492-B26] Prélot L , David JK, Lin A. Splicing neoantigen prediction is sensitive to methodological difference. bioRxiv, 10.1101/2025.09.10.674685, 2024, preprint: not peer reviewed.

[btaf492-B27] Richman LP , Vonderheide RH, Rech AJ. Neoantigen dissimilarity to the Self-Proteome predicts immunogenicity and response to immune checkpoint blockade. Cell Syst 2019;9:375–82.e4. 31606370 10.1016/j.cels.2019.08.009 PMC6813910

[btaf492-B28] Richters MM , Xia H, Campbell KM et al Best practices for bioinformatic characterization of neoantigens for clinical utility. Genome Med 2019;11:56. 31462330 10.1186/s13073-019-0666-2 PMC6714459

[btaf492-B29] Rudnick PA , Markey SP, Roth J et al A description of the clinical proteomic tumor analysis consortium (CPTAC) common data analysis pipeline. J Proteome Res 2016;15:1023–32. 26860878 10.1021/acs.jproteome.5b01091 PMC5117628

[btaf492-B30] Ruggles KV , Tang Z, Wang X et al An analysis of the sensitivity of proteogenomic mapping of somatic mutations and novel splicing events in cancer. Mol Cell Proteomics 2016;15:1060–71. 26631509 10.1074/mcp.M115.056226 PMC4813688

[btaf492-B31] Schäfer RA , Guo Q, Yang R. ScanNeo2: a comprehensive workflow for neoantigen detection and immunogenicity prediction from diverse genomic and transcriptomic alterations. Bioinformatics 2023;39:btad659. 37882750 10.1093/bioinformatics/btad659 PMC10629934

[btaf492-B32] Schumacher TN , Schreiber RD. Neoantigens in cancer immunotherapy. Science 2015;348:69–74. 25838375 10.1126/science.aaa4971

[btaf492-B33] Sharon D , Tilgner H, Grubert F et al A single-molecule long-read survey of the human transcriptome. Nat Biotechnol 2013;31:1009–14. 24108091 10.1038/nbt.2705 PMC4075632

[btaf492-B34] Smart AC , Margolis CA, Pimentel H et al Intron retention is a source of neoepitopes in cancer. Nat Biotechnol 2018;36:1056–8. 30114007 10.1038/nbt.4239 PMC6226333

[btaf492-B35] Tappeiner E , Finotello F, Charoentong P et al TIminer: NGS data mining pipeline for cancer immunology and immunotherapy. Bioinformatics 2017;33:3140–1. 28633385 10.1093/bioinformatics/btx377 PMC5870678

[btaf492-B36] Wang T-Y , Wang L, Alam SK et al ScanNeo: identifying indel-derived neoantigens using RNA-Seq data. Bioinformatics 2019;35:4159–61. 30887025 10.1093/bioinformatics/btz193

[btaf492-B37] Wei Z , Zhou C, Zhang Z et al The landscape of tumor fusion neoantigens: a Pan-Cancer analysis. iScience 2019;21:249–60. 31677477 10.1016/j.isci.2019.10.028 PMC6838548

[btaf492-B38] Wen B , Wang X, Zhang B. PepQuery enables fast, accurate, and convenient proteomic validation of novel genomic alterations. Genome Res 2019;29:485–93. 30610011 10.1101/gr.235028.118 PMC6396417

[btaf492-B39] Wickland DP , McNinch C, Jessen E et al Comprehensive profiling of cancer neoantigens from aberrant RNA splicing. J Immunother Cancer 2024;12:e008988. 38754917 10.1136/jitc-2024-008988 PMC11097882

[btaf492-B40] Wood MA , Nguyen A, Struck AJ et al neoepiscope improves neoepitope prediction with multivariant phasing. Bioinformatics 2020;36:713–20. 31424527 10.1093/bioinformatics/btz653

[btaf492-B41] Zhang B , Whiteaker JR, Hoofnagle AN et al Clinical potential of mass spectrometry-based proteogenomics. Nat Rev Clin Oncol 2019;16:256–68. 30487530 10.1038/s41571-018-0135-7 PMC6448780

[btaf492-B42] Zhang J , Mardis ER, Maher CA. INTEGRATE-neo: a pipeline for personalized gene fusion neoantigen discovery. Bioinformatics 2017;33:555–7. 27797777 10.1093/bioinformatics/btw674 PMC5408800

[btaf492-B43] Zhang Z , Zhou C, Tang L et al ASNEO: identification of personalized alternative splicing based neoantigens with RNA-seq. Aging (Albany NY) 2020;12:14633–48. 32697765 10.18632/aging.103516 PMC7425491

[btaf492-B44] Zhu C , Liu LY, Ha A et al MoPepGen: Rapid and comprehensive identification of non-canonical peptides. bioRxiv, 10.1101/2024.03.28.587261, 2024, preprint: not peer reviewed.

