# Supplementary material for: ImmunoPepper: extracting personalized peptides from complex splicing graphs
Source: Bioinformatics. 2025 Oct 9;42(1):btaf492. doi: 10.1093/bioinformatics/btaf492 (PMC12777967; doi:10.1093/bioinformatics/btaf492)
Supplement: btaf492_Supplementary_Data [file btaf492_supplementary_data.pdf]

1 Supplementary Results

1.1 Validation rate of proteomics experiments

Estimation of the validation rate in proteomics is a difficult task. On the one hand, MS technologies like DDA involve peptide-space sampling (Verheggen *et al.*, 2017; Ruggles *et al.*, 2016) which leads to non-reproducibility of measurements between replicates from a given sample. On the other hand, computational analysis of mass spectrometry data is a heterogeneous research field, with a large panel of available algorithms (Nesvizhskii, 2010). Proteogenomics analyses build an additional layer of complexity (Nesvizhskii, 2014). These rely on the building of a database of computationally-derived candidate neoepitopes. These rare peptides tend to be especially hard to detect in mass spectrometry datasets as they can lose signal to both major proteins and their minor products (less frequent post-translational modifications) in the target-decoy discovery framework (Lin *et al.*, 2021).

To understand how high the expected validation rate of proteogenomics experiments can be, we propose some estimates based on a study which protocol is close enough to our work (Ruggles *et al.*, 2016). The estimated validation percent between 0.014% and 2.99% can be found in Supplementary Table 1. The authors use iTRAQ data, similar to our study, as well as label-free global proteomics data. Target-target decoy discovery is performed with the X! Tandem (Craig and Beavis, 2004) database search engine (*SNS* is a target-decoy discovery framework). Both studies build the proteogenomics database by concatenation of the reference and the neoepitope candidate peptides (searching against the neoepitope candidates only, as performed by (Li *et al.*, 2024) would not control the FDR). The FDR that the authors use is 1%, while we chose an FDR of 5%. The authors perform peptide-FDR while we perform PSM-FDR. The two last features make the (Ruggles *et al.*, 2016) analysis more stringent. However, cancer-specificity criteria in our study are highly stringent compared to the ones applied in (Ruggles *et al.*, 2016), reducing the number of candidates to be matched against MS data. The authors obtain tumor-specific variants by filtering out all germline variants from the somatic variant calls and by filtering out splice junctions coordinates with previously annotated boundaries. In this work, we exclude from the set of neoepitope candidates any normal peptide translated from a panel of normals, after the insertion of the tumor germline variants (and without germline variant application). Moreover, the splicing analysis of our study relies on a homogeneously processed panel of normal and cancer RNA-seq datasets. Both studies have additional filters against translational databases and use hg19 as a reference for the translation of the peptides, which makes the set splicing-derived candidates comparable. Finally, the authors included all RNA-Seq junctions in the MS database regardless of the read support, while we selected the candidates based on a combination of the support in the sample and the cancer cohort, making our approach more stringent.

We then looked at the validation rates from the (Pan *et al.*, 2023) study. The validation percentages between 0.11 % and 0.29% can be found in Supplementary Table 2. The authors implement a very different experimental protocol, as the MS analysis of splicing-derived neoepitopes was performed without assessing tumor specificity (in-silico screening module). A stringent cancer junction coverage criteria was set in the sample with a threshold of at least 10 reads. Importantly the study was performed on Immunopeptidomics data while our study uses proteomics data. The candidates were searched against the MS data with MSGF+ (Kim and Pevzner, 2014) a target-decoy discovery tool.

Neoepitope candidate origin	Cancer subtype	Novel candidate peptides	Novel peptides detected by MS	Validation percentage
Splicing-derived	Basal-like*	364815	66	0.018%
Splicing-derived	Luminal-like*	478793	67	0.014%
SNV-derived	Basal-like*	20435	610	2.99 %
SNV-derived	Luminal-like*	18296	496	2.71 %

Table 1. Estimation of proteomics validation rates from (Ruggles et al., 2016). (*) Luminal and basal-like breast-cancer-patient-derived xenografts. (**) These candidate sets were included for the matching against the MS data. The data was extracted from Figure 2 and 4 of (Ruggles et al., 2016).

Neoepitope candidate origin	Cell type	Novel candidate 9-mers	Novel 9-mers detected by MS	Validation percentage
Splicing-derived	JeKo-1*	230	78332	0.29%
Splicing-derived	B-LCL-S1**	178	103032	0.17%
Splicing-derived	B-LCL-S2**	85	79742	0.11%

Table 2. Estimation of proteomics validation rates from (Pan et al., 2023). (*) lymphoma cancer cell line (**) B lymphoblastoid cell lines. The data was extracted from Figure 2 b of (Pan et al., 2023).

1.2 Design choices of the ImmunoPepper implementation

1.2.1 Potential combinatorial explosion of mutations for the case of 63 OV and BRCA samples

Assuming that a gene is not spliced, it can be assimilated to a string on which the somatic mutations are combinatorially inserted. If we have n somatic mutations for a given gene, there are 2^n combinations of somatic mutations and 2^n different sequences. In the case of OV and BRCA samples of this study, between 78 and 229 somatic mutations per sample were input to the *ImmunoPepper* software tool. Gene-wise, this led to the combination of at most three somatic mutations per gene. We did not encounter a combinatorial explosion in a highly mutated region. This is due to two factors: the cancer type (ICGC/TCGA Pan-Cancer Analysis of Whole Genomes Consortium, 2020) and the stringent variant calling (Kahles *et al.*, 2018).

1.2.2 Case of other cancer types and larger cancer cohorts

(Alexandrov *et al.*, 2013) analyzed the prevalence of somatic mutations across 30 human cancer types. 28 cancer types had, in the cohorts studied, a median number of somatic mutations per megabase (Mb) less or equal to 10, while melanoma and lung squamous cancers had medians above 10. Seven cancer types had patients with 100 or more somatic mutations per Mb. The uterus, stomac, colorectum, and melanoma cancer cohorts had a significant tail of patients with between 100 to $\approx$ 400 somatic mutations per Mb (estimate from text and Figure 1 (Alexandrov *et al.*, 2013)). In the case of melanoma cancer, another study (Hodis *et al.*, 2012) found a lower-upper quartile range of 8.0 to 24.9 mutations per Mb in melanoma samples. Taking 400 somatic mutations per Mb as an estimate for the highly mutated outlier patients, we can estimate 0.0004 somatic mutations per base. This gives in expectation 0.048 mutations for 120 bases, which is the median length of an exon (Mokry *et al.*, 2010), or 0.4 mutations for 1000 bp, for extremely long exons, which represent less than 0.5% of human exons (Mokry *et al.*, 2010).

As the mutations are non-uniformly distributed over the genome, the gene-level view is important to consider. At the gene level (Ding *et al.*, 2014) analyzed genes with statistically significantly higher mutation rates than expected based on the background mutation rate in metastatic melanoma. Among those, were BRAF, NRAS, CDKN2A and EPHA3 with a mutation rate of 110 to 350 mutations per Mb.

The exon-level rate is more difficult to estimate from previous studies. Mutations tend to cluster in hotspot mutation regions. The BRAF gene mutation is highly prevalent in melanoma (Hayward *et al.*, 2017). It has been found that 97% of the alterations in the BRAF genes occur in exon 15 (Ihle *et al.*, 2014), with 90% being the p.V600E mutant (Ihle *et al.*, 2014). This implies that the many variants reported for the highly mutated regions between exons 11 and 15 of BRAF variants can only be observed in very large cohorts of patients; the mutation structure of this region has been described by (Roa *et al.*, 2024). However, we can compute a bound from the computational side. Although at the limit, our approach could generate up to 1000 variant-combinations per exon-pair, corresponding to approximately 10 individual variants. Seeing 10 somatic variations in an exon-pair, even if this exon pair is 2000 bases long, would amount to a somatic mutation rate of 5000 mutations per megabase, which is orders of magnitude above even the observed outlier samples. In this context, it is important to note that we only need to combine variants occurring in the same sample.

1.2.3 Features to control the combinatorial burden

The ImmunoPepper software, offers features that help control memory strain from a high combination number. On the one hand, *ImmunoPepper* can exclude junction, bi, or tri-exon peptides, as well as k-mers to candidates on-the-fly. This operation reduces both the memory and the size of the output (I/O). For example, the software supports the filtering of k-mers, against a database of candidates that should not be output. In our analysis, we chose to use the UniProt (UniProt Consortium, 2023) database. This database could contain some common somatic variants. On-the-fly filtering can also be performed against a set of junction coordinates. Besides, upon translation, peptides that are synonymous with the annotated peptides are not further processed. To further reduce the complexity of the output: k-mers from mutations (and alternative splicing are made unique whenever possible). Note that to reduce the complexity of the alternative splicing paths, the user can enable the merging of exons spanning the same junctions into the the longest-spanning sequence. On the other hand, *ImmunoPepper* implements parallelization that aims at reducing the memory impact. The genes can be processed in multiprocessing pools, with the size of the gene batch being a user-set parameter. The processing with *imap* was chosen for its capacity to control memory better on a iterable which can be as big as the number of genes in the genome and its ability to retrieve the results as soon as they are ready for saving to disk. The saving is also performed in a distributed way with possible writing of partitions.

2 Supplementary Methods

2.1 ImmunoPepper Outputs

ImmunoPepper returns peptides generated from 2 or 3 exons and containing one or two junctions respectively. Besides, *ImmunoPepper* returns *k-mers* with k specified by the user. *ImmunoPepper* distinguishes between *junction k-mers* and *junction k-mers* defined in the following.

2.1.1 Notations

In the following, Θ is defined as the function mapping the RNA to the amino-acid alphabet.

S is a string of exactly k amino acids (AAs): $S = \Theta[((g_b, g_d), (g_e, g_f))]$.

$((g_b, g_d), (g_e, g_f))$ is a sub-string of the exon pair (v_i, v_j). It results from the concatenation of (g_b, g_d) and (g_e, g_f), which

are sub-strings of the exon (g_a, g_d) and (g_e, g_h) respectively. This means: $g_a \leq g_b < g_d < g_e \leq g_f < g_h$.

A junction is defined as the pair of the last exon coordinate of the left exon and the first exon coordinate of the right exon $J_{e,b} := (g_d, g_e)$ and $g_d < g_e$.

2.1.2 Concept of *junction k-mer*

We denote as $\mathcal{K}^{\mathcal{J}}$ the set of all k-mers of sequence exactly S, originating from 2 different exons, i.e. including a junction (in some cases originating from 3 exons). These are called *junction k-mers*. We can write:

$$\mathcal{K}^{\mathcal{J}} = \{((g_b, g_d), (g_e, g_f)) | \forall (g_b, g_d, g_e, g_f), \\ \Theta[((g_b, g_d), (g_e, g_f))] = S\}$$

2.1.3 Concept of *segment k-mer*

We denote $\mathcal{K}^{\mathcal{S}}$ the set of all k-mers of sequence exactly S, originating from genomic coordinates which do not include a junction. These are called *segment k-mers*. We can write:

$$\mathcal{K}^{\mathcal{S}} = \{(g_b, g_d) | \forall (g_b, g_d), \Theta[(g_b, g_d)] = S\}$$

2.2 Expression calculation used in *ImmunoPepper*

The expression metrics output by *ImmunoPepper* are as follows:

Junction expression : If edge expression data is provided with the splice-graph, we output the expression of the edge to which this junction corresponds.

Segment expression : If segment expression data is provided with the splice-graph, we calculate the average segment expression for each generated peptide. We follow the definition of segments in (Kahles *et al.*, 2016): A vertex v_i is composed from segments $s_{i,q}$ through $s_{i,r}$, if $v_i = s_{i,q} \circ_{i,r}$. Hereby, $\circ$ denotes the concatenation of segment positions. The expression counts and segment length for segment $s_{i,q}$ are $EC_{i,q}$ and $SL_{i,q}$, respectively. The length for vertices v_i and v_j are VL_i and VL_j, with $VL_i := \sum_{k=q_i}^{r_i} SL_{i,k}$.

The segment expression for a translated vertex pair (v_i, v_j) is defined as:

$$SE_{ij} := \frac{1}{VL_i + VL_j} (\sum_{k=q_i}^{r_i} SL_{i,k} EC_{i,k} + \sum_{k=q_j}^{r_j} SL_{j,k} EC_{j,k})$$

Each generated peptide sequence is associated with its expression metadata.

2.3 Cohorts used in the experiments

The UniProt (UniProt Consortium, 2023) database UP000005640 was downloaded from https://www.UniProt.org/proteomes /UP000005640. 32 TCGA-OV and 31 TCGA-BRCA samples from the TCGA cohorts were selected as in (Kahles *et al.*, 2018) focusing on CPTAC (Rudnick *et al.*, 2016) data availability. The GTEx (Lonsdale *et al.*, 2013) cohort included all 3'233 samples used in (Kahles *et al.*, 2018).

2.4 Start-to-finish neoepitope candidate generation and filtering with the *re-quant* and *all-frames* pipelines.

For all *junction 9-mers* derived from the TCGA cancer samples and their *mutated* version (either bearing a germline variant, a somatic mutation or both), the normal GTEx background 9-mers are filtered following two different setups: the *re-quant* and the *all-frames* pipelines.

2.4.1 Notations

Let $E_{\lambda,\text{Normal}} \in \mathbb{R}^n$, $E_{\lambda,\text{Normal}} = [e_1 \; e_i \; \cdots \; e_n]$ be defined as the normalised expression vector taken across n samples for the normal cohort for the k-mer sequence λ (either a junction k-mer or a segment k-mer).

The vectors for junction expression and segment expression (as defined in 2.2) will be denoted $E^{\gamma}_{\lambda,\text{Normal}}$ and $E^{\sigma}_{\lambda,\text{Normal}}$, respectively.

For the count vector $E^{\gamma}_{\lambda,\text{Normal}}$, if no sample is expressed in the normal cohort, we can write:

$$E^{\gamma}_{\lambda,\text{Normal}} = \mathbf{0}_n \iff E^{\gamma}_{\lambda,\text{Normal}} \in \mathbb{R}^n, \nexists e_j > 0, \forall j \in \{1...n\}$$

The same applies to the segment expression vector $E^{\sigma}_{\lambda,\text{Normal}}$.

Let set $k = 9$. Let define $\mathcal{K}^{\mathcal{J}}$ and $\mathcal{K}^{\mathcal{S}}$ the sets of *junction 9-mers* with sequence S and *segment 9-mers* with sequence S, respectively, as defined in the 2.1.2 and 2.1.3. We will index the latter as $\mathcal{K}^{\mathcal{J}}_{\text{Cohort}}$ and $\mathcal{K}^{\mathcal{S}}_{\text{Cohort}}$ for the cohorts from which the *k-mers* originate. We will consider a cancer and a normal cohort.

2.4.2 Filtering goals

The cancer cohort filtering aims at setting the thresholds for acceptation of *junction 9-mer* candidates based in the expression in the sample of interest or in the cancer cohort. This step has been described in the Methods.

Besides, the *junction 9-mers* are filtered based on a normal filtering based on the GTEx cohort. Exclusion criteria are as follows: (a) a synonymous 9-mer AA of sequence S overlaps an exon-exon junction positions expressed in the background cohort (b) a synonymous 9-mer AA of sequence S generated from a non junction-overlapping sequence has any of its segment positions expressed in the background cohort.

2.4.3 Processing for the generation of the foreground set of peptides

Foreground peptides were generated from splicing junctions with or without personal variation. Splicing junction alone

or in combination with: (1) germline variation, (2) somatic mutation or (3) both, were considered. The foreground peptides were translated following GENCODE annotated reading frames. Peptide 9-mers present in the UniProt (UniProt Consortium, 2023) database were suppressed on-the-fly from the output.

The input of the software were:

- A splicing graph generated with *SplAdder* (Kahles *et al.*, 2016) from the TCGA cohort samples.
- A count file generated with *SplAdder* (Kahles *et al.*, 2016). The identity of the cohorts differs between the *re-quant* and *all-frames* pipelines.
- The germline variant calling VCFs and mutation MAFs files from the TCGA cohort samples.
- The GENCODE annotation file *gencode.v19.annotation.hs37d5_chr* (Harrow *et al.*, 2012), consistently with the annotation version of the input graphs.

Subsequently to the generation of the 9-mers candidates, candidates derived from the translation of annotated transcripts were removed. This included 9-mers from annotated transcripts with germline variant insertion.

2.4.4 Foreground set of peptides in the *re-quant* pipeline

In this pipeline, the count file provided to the software contained the GTEx cohort counts matched to the TCGA graph. The file was generated by quantifying the splicing graph from the TCGA cohort based on the alignment files from the GTEx cohort with the *SplAdder* software (Kahles *et al.*, 2016). Thereby, the *re-quant* pipeline does not require a separate background set of peptides. Because each position containing a germline variant was quantified against a large cohort of normal, it prevented reporting germline variant peptides as tumor-specific candidates.

2.4.5 Foreground set of peptides in the *all-frames* pipeline

In this pipeline, the count file provided to the software contained the TCGA cohort counts matched to the TCGA graph.

2.4.6 Processing of the background set of peptides: Case of the *all-frames* pipeline

Background peptides were generated from the combination of 2 or 3 exons with or without personalization. By inserting the germline variation sample-wise into the GTEx background peptides, we achieved a personalized background for each sample and prevented the germline variant peptides from being reported as tumor-specific candidates. The peptides were translated following 3 reading frames, which constitute all possible reading frames for a strand. Peptide 9-mers present in the UniProt (UniProt Consortium, 2023) database were suppressed on-the-fly from the output.

The input of the software were:

- A splicing graph generated with *SplAdder* (Kahles *et al.*, 2016) from the GTEx cohort samples.
- No count file, meaning that any peptide present in any sample of the GTEx graph would be generated.
- The germline variant calling VCFs from the TCGA cohort samples. This allows for application of foreground germline variation to the background set of peptides.
- The GENCODE annotation file *gencode.v19.annotation.hs37d5_chr* (Harrow *et al.*, 2012), consistently with the annotation version of the input graphs.

Subsequently to the generation of the 9-mers candidates, candidates derived from the translation of annotated transcripts were removed. This included 9-mers from annotated transcripts with germline variant insertion.

2.4.7 Cancer filtering applied to the foreground set of peptides

To ensure the relevance of each candidate in cancer biology, the following filtering criteria was applied to the foreground set: A given 9-mer was retained if it was expressed in the RNA-seq of the cancer sample with any read count or if the sum of the normalized reads across the 32 TCGA-OV and 31 TCGA-BRCA samples was at least 20. This criteria matches exactly the cancer filtering criteria implemented in (Kahles *et al.*, 2018) and was applied in both the *re-quant* and *all-frames* pipelines.

2.4.8 Normal filtering algorithm applied in the *re-quant* pipeline

The *re-quant* filtering setup keeps *junction 9-mer* candidates translated via novel propagated reading frames and from novel junctions.

For each 9-mer $\lambda \in \mathcal{K}^{\mathcal{J}}_{\text{Cancer}}$, there exist an expression vector $E^{\gamma}_{\lambda,\text{Normal}}$ or $E^{\gamma}_{\sigma,\text{Normal}}$ (as defined in 2.1.2, 2.1.3 and 2.4.1) derived from the quantification of the cancer graph based on the normal reads (See 2.4.4).

The filtered *junction 9-mer* set was computed as:

$$T := \{\lambda \in \mathcal{K}^{\mathcal{J}}_{\text{Cancer}} | E^{\gamma}_{\lambda,\text{Normal}} = \mathbf{0}_n\} \bigcap \{\lambda \in \mathcal{K}^{\mathcal{J}}_{\text{Cancer}} | E^{\sigma}_{\lambda,\text{Normal}} = \mathbf{0}_n\}$$

2.4.9 Normal filtering algorithm applied in the *all-frames* pipeline

The *all-frames* filtering setup keeps *junction 9-mer* candidates translated from novel junctions.

For each 9-mer $\lambda \in \mathcal{K}^{\mathcal{J}}_{\text{Normal}}$ (resp. $\lambda \in \mathcal{K}^{\mathcal{S}}_{\text{Normal}}$), there exist an expression vector $E^{\gamma}_{\lambda,\text{Normal}}$ (resp. $E^{\sigma}_{\lambda,\text{Normal}}$) (as defined in 2.1.2, 2.1.3 and 2.4.1) derived from the normal cohort (See 2.4.6).

In this pipeline, the candidate 9-mers $\lambda \in \mathcal{K}^{\mathcal{J}}_{\text{Cancer}}$ generated from the cancer graph cannot be directly quantified in the normal cohort, as the cancer and the normal graph are two separate data structures. A set difference approach between cancer and normal 9-mers was

used.

The filtered *junction 9-mer* set was computed as:

$$T := \{\lambda \in \mathcal{K}^{\mathcal{J}}_{\text{Cancer}} | \lambda \in \mathcal{K}^{\mathcal{J}}_{\text{Normal}} \Rightarrow E^{\gamma}_{\lambda,\text{Normal}} = \mathbf{0}_n\} \bigcap \{\lambda \in \mathcal{K}^{\mathcal{J}}_{\text{Cancer}} | \lambda \in \mathcal{K}^{\mathcal{S}}_{\text{Normal}} \Rightarrow E^{\sigma}_{\lambda,\text{Normal}} = \mathbf{0}_n\}$$

A correction was performed by quantifying lowly expressed 9-mers (less than 2 reads) based on the reads counts in the BAM alignement files instead of the read counts in the *SplAdder* (Kahles *et al.*, 2016) graph.

2.5 MS validation with the *PepQuery* and the *Subset-Neighbor-Search* approaches

The tryptic-junction peptides' presence in the MS data was searched both with the *PepQuery* (Wen *et al.*, 2019) engine and the *Subset-Neighbor-Search* (Lin *et al.*, 2021) approach.

2.5.1 Experimental design for *PepQuery*

For the *PepQuery* search two options were compared (1) keeping the peptides matching the spectra better than the reference peptides and the shuffled peptides (2) keeping the peptides matching the spectra better than the reference peptides, the shuffled peptide and the post-translational modified reference peptides (ptm-ref-p). We believe that (1) is better suited than (2) for the discovery of neoantigens because ptm-ref-p inclusion creates a competition between minor by-products of major components (ptm-ref-p) and major products of minor proteins (neoantigens) (Duncan *et al.*, 2010). This can lead to false positive matches between ptm-ref-p and the spectra, which, because *PepQuery* does not control the FDR like target-decoy competition (TDC) approaches do, would falsely reduce the detection of neoantigens. In this paper, we run *PepQuery* with all default parameters and report (1). Note, we could have adjusted the parameters to get (1) as final output.

2.5.2 Experimental design for the *Subset-Neighbor-Search*

For the *Subset-Neighbor-Search*, the tryptic-junction peptides were used as the target peptides. The *neighbor* peptides (Lin *et al.*, 2021) were derived from the target peptides against the reference UniProt database. Then the target and the *neighbor* peptides were searched jointly with the Tide search engine (Diament and Noble, 2011) within the *Crux* toolkit (Kertesz-Farkas *et al.*, 2023) in each of the MS fractions. All default parameters were used except `-precursor-window 40` and `-top-match 1`. The search results were concatenated, the target peptide set extracted and target-decoy competition was performed. The false discovery rate (FDR) was estimated using the `assign-confidence` function within the *Crux* toolkit (Kertesz-Farkas *et al.*, 2023) with default parameters. Specifically, peptide-spectrum-match-level (PSM-level) FDR was estimated and peptides with PSMs passing a 5% PSM-level FDR were reported as detected. A peptide-level FDR calculation was also tested with the *Crema* (Lin *et al.*, 2024) method. However, it was not found suitable due to the very limited number of input candidates.

2.5.3 Negative control experiment

The 2 and 3 exons were translated following all possible reading frames (no personalisation with somatic mutation or germline variants was applied) and split into 9-mers. All 9-mer candidates which follow a reading frame found in the annotation were filtered-out to produce a set of 9-mers translated in the "wrong frame". Additionally, all 9-mers present in UniProt were removed from the set. The candidates 9-mers were searched in the MS data of the TCGA samples. Because the MS-validation rate is highly driven by the number of peptides included in the library for matching, sub-sampling was performed. We refer to the original number of annotated-frame candidates from the *re-quant* or *all-frames* experiment as the "library size to reproduce". The 9-mers were mapped back to their longer peptide context and tryptic-digestion was performed. The library obtained consists of "tryptic-junction peptides translated from wrong frames". These peptides were sampled to get a subset of size that matches the library size to reproduce. The sampled peptides were searched in the TGCA Mass Spectrometry data following the *PepQuery* and the *Subset-Neighbor-Search* methods described in the manuscript. The sampling and search was repeated 10 times for each sample.

3 Supplementary Figures

a

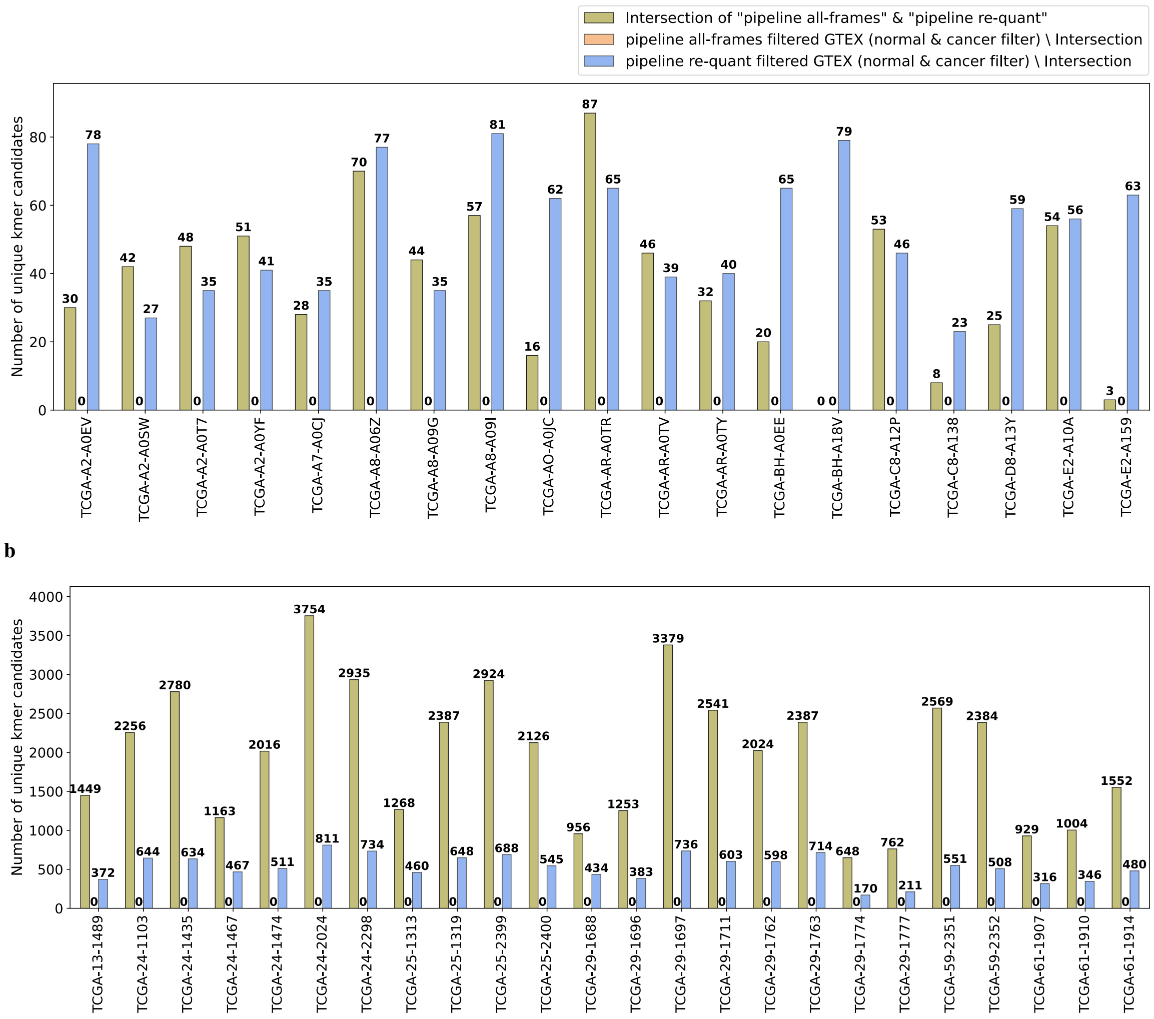

Fig. 1: **Exploration of the two candidate generation methods supported by ImmunoPepper.** Green: Number of 9-mers present in the intersection between the *re-quant* and the *all-frames* pipeline outputs. Blue: Pipeline *re-quant* output minus the previously defined intersection. Orange: Pipeline *all-frames* output minus the intersection. Results for the pipelines are presented after cancer-level and normal-level filtering. A: BRCA samples. B: Ovarian samples.

Pipeline/Tool	Germline context	Somatic variants	Transcript variants	Splicing graph	RF propagation on transcript
ImmunoPepper	✓	SNVs/ INDELs	AS via SplAdder	✓	✓
Comp. An. Spli.	✓	SNVs/ INDELs	AS via SplAdder	✓	✗
Splice2neo	✗	SNVs/ INDELs	AS (restricted to AS from somatic mutations) via SplAdder, LeafCutter	✓	✗
RegTools	✗	SNVs/ INDELs	AS (restricted to AS from somatic mutations)	✗	N/A
SNAF	✗	✗	AS via AltAnalyze	✗	N/A
IRIS	✗	✗	AS via rMATS	✗	N/A
Neosplice	✗	SNVs/ INDELs (not jointly with AS)	AS	✓	✗
ASNEO	✗	✗	AS	✗	N/A
ProTECT	✗	SNVs/ INDELs	Gene fusions	✗	N/A
Neoepiscope	✓	SNV/ INDELs/ Phased	✗	✗	N/A
pVACTools	✓	SNVs/ INDELs	Gene fusions	✗	N/A
ScanNeo	✗	Large-scale INDELs	✗	✗	N/A
Epidisco (PVG trial)	✓	SNVs/ INDELs (via STAR)	Some splice junctions if short-range from somatic variants	✗	N/A
TSNAD, TSNAD.V2	✗	SNVs/ INDELs	Gene fusions	✗	N/A
MuPeXI	✗	SNVs/ INDELs	✗	✗	N/A
INTEGRATE-Neo	✗	✗	Gene fusions	✗	N/A
TIminer	✗	SNVs	✗	✗	N/A
CustomProDB	✗	SNVs/ INDELs (not jointly with AS)	Some splice junctions (e.g., TopHat)	✗	N/A

Table 3. Comparison of ImmunoPepper with other tools or computational pipelines that generate putative neo-epitopes, in terms of sensitivity for detecting plausible peptides. ImmunoPepper extracts junctions from a splice graph and personalizes peptides with germline context and all combinations of somatic mutations, in contrast to all competitors. ImmunoPepper builds on the analysis from (Kahles et al., 2018), here denoted as "Comp. An. Spli". RF: Reading frames. References to compared neoepitope Pipelines/Tools: Splice2neo (Lang et al., 2024), RegTools (Cotto et al., 2023), SNAF (Li et al., 2024), IRIS (Pan et al., 2023), Neosplice (Chai et al., 2022), ASNEO (Zhang et al., 2020), ProTECT (Rao et al., 2020), Neoepiscope (Wood et al., 2020), pVACTools (Hundal et al., 2020), ScanNeo (Wang et al., 2019), Comp. An. Spli. (Kahles et al., 2018), Epidisco (PVG trial) (Rubinsteyn et al., 2018), TSNAD (Zhou et al., 2017), TSNAD.V2 (Zhou et al., 2021), MuPeXI (Bjerregaard et al., 2017), INTEGRATE-Neo (Zhang et al., 2017), TIminer (Tappeiner et al., 2017), CustomProDB (Wang and Zhang, 2013). AS tools: SplAdder (Kahles et al., 2016), LeafCutter (Li et al., 2018), rMATS (Shen et al., 2014; Wang et al., 2024), STAR (Dobin et al., 2013), TopHat (Trapnell et al., 2009), AltAnalyze (Alt, 2018).

Pipeline/Tool	Presented Transcriptome BG	Integrated Transcriptome BG	Proteome BG	RNA expr.	Presented MHC binding	Integrated MHC binding	Presented MS val.	Integrated MS val.
ImmunoPepper	GTEx, Translated annotation	✓	UniProt DB	✓	MHCflurry, NetMHC, NetMHCpan, NetMHCIIpan, NetMHCcons, IedbMhcClass1, IedbMhcClass2	✓	PepQuery, Subset Neighbor Search	✓(PepQuery)
Comp. An. Spli.	GTEx, Translated annotation	✗	UniProt DB	✓	NetMHC	✗	MS-GF+	✗
Splice2neo	GTEx, Translated annotation	✓	✗	✓	NetMHCpan, MixMHCpred, NetMHCIIpan, MixMHC2pred via NeoFox	✗	✗	✗
RegTools	GTEx, Annotated junctions	✗	✗	External tool	✗	✗	✗	✗
SNAF	GTEx	✓	UniProt DB	✓	MHCflurry	✓	MaxQuant/ Andromeda	✗
IRIS	GTEx	✓	✗	✓	NetMHC	✓	MS-GF+	✓
Neosplice	Matched Normal Samples, Translated annotation	✓	✗	External tool	NetMHCpan	✓	Bruker DataAnalysis LibraryEditor	✗
ASNEO	GTEx, Annotated junctions	✓	✗	External tool	NetMHCpan	✓	Comet/Percolator	✗
ProTECT	✗	✗	✗	✓	IedbMhcClass1 , IedbMhcClass2	✓	✗	✗
Neoepiscope	Matched Normals	✓	Matched normal proteins	External tool	MHCflurry, MHCnuggets, NetMHCpan, NetMHCpanII	✓	✗	✗
pVACTools	Matched Normals	✓	✗	External tool	NetMHC, NetMHCpan, NetMHCcon, Pickpocket, SMM, SMMPMBEC, MHCflurry, NetMHCpanII, SMM-align, NN-align, MHCnuggets	✓	✗	✗
ScanNeo	✗	✗	✗	External tool	NetMHCpan, NetMHC	✓	X!Tandem, MS-GF+, OMSSA (intersection 3 tools)	✗
Epidisco (PVG trial)	✗	✗	✗	✓	MHCflurry, NetMHC, NetMHCpan, NetMHCIIpan, NetMHCcons, IedbMhcClass1, IedbMhcClass2	✓	✗	✗
TSNAD, TSNAD.V2	✗	✗	✗	External Tool	DeepHLApan, MHCflurry, NetMHCpan (intersection 3 tools)	✓	✗	✗
MuPeXI	✗	✗	✗	External tool	NetMHCpan	✓	✗	✗
INTEGRATE-Neo	✗	✗	✗	External tool	NetMHC	✓	✗	✗
TIminer	✗	✗	✗	External tool	NetMHCpan	✓	✗	✗
CustomProDB	✗	✗	✗	✓	✗	✗	✗	✗

Table 4. Comparison of filtering mechanisms supported by *ImmunoPepper* with other tools or computational pipelines for generation of putative neo-epitopes. *ImmunoPepper* builds on Comp. An. Spli. (Kahles et al., 2018). Background, expr.: expression, val.: validation. Neoepitope Pipelines/Tools: SNAF (Li et al., 2024), IRIS (Pan et al., 2023), Neosplice (Chai et al., 2022), ASNEO (Zhang et al., 2020), ProTECT (Rao et al., 2020), Neoepiscope (Wood et al., 2020), pVACTools (Hundal et al., 2020), ScanNeo (Wang et al., 2019), Comp. An. Spli. (Kahles et al., 2018), Epidisco (PVG trial) (Rubinsteyn et al., 2018), TSNAD (Zhou et al., 2017), TSNAD.V2 (Zhou et al., 2021), MuPeXI (Bjerregaard et al., 2017), INTEGRATE-Neo (Zhang et al., 2017), TIminer (Tappeiner et al., 2017), CustomProDB (Wang and Zhang, 2013). MHC-binding tools: NeoFox (Lang et al., 2021), MixMHCpred (Bassani-Sternberg et al., 2017; Gfeller et al., 2023), MixMHC2pred (Gfeller et al., 2018) MHCflurry (O'Donnell et al., 2020), MHCnuggets (Shao et al., 2020), DeepHLApan (Wu et al., 2019), NetMHCpan (Jurtz et al., 2017), NetMHCpanII (Jurtz et al., 2017), NetMHCcons (Karosiene et al., 2012), SMMPMBEC (Kim et al., 2009), Pickpocket (Zhang et al., 2009), NN-align (Nielsen and Lund, 2009), NetMHC (Lundegaard et al., 2008; Andreatta and Nielsen, 2016), SMM-align (Nielsen et al., 2007), SMM (Peters and Sette, 2005), IedbMhcClass1 (IED, 2005), IedbMhcClass2 (IED, 2005). MS/MS analysis tools/ frameworks: Subset Neighbor Search (Lin et al., 2021), PepQuery (Wen et al., 2019), MS-GF+ (Kim and Pevzner, 2014), Comet (Eng et al., 2013), Andromeda (Cox et al., 2011), MaxQuant (Cox and Mann, 2008), Percolator (Käll et al., 2007), X!Tandem (Craig and Beavis, 2004), OMSSA (Geer et al., 2004), Bruker DataAnalysis LibraryEditor (bruker software, 2024).

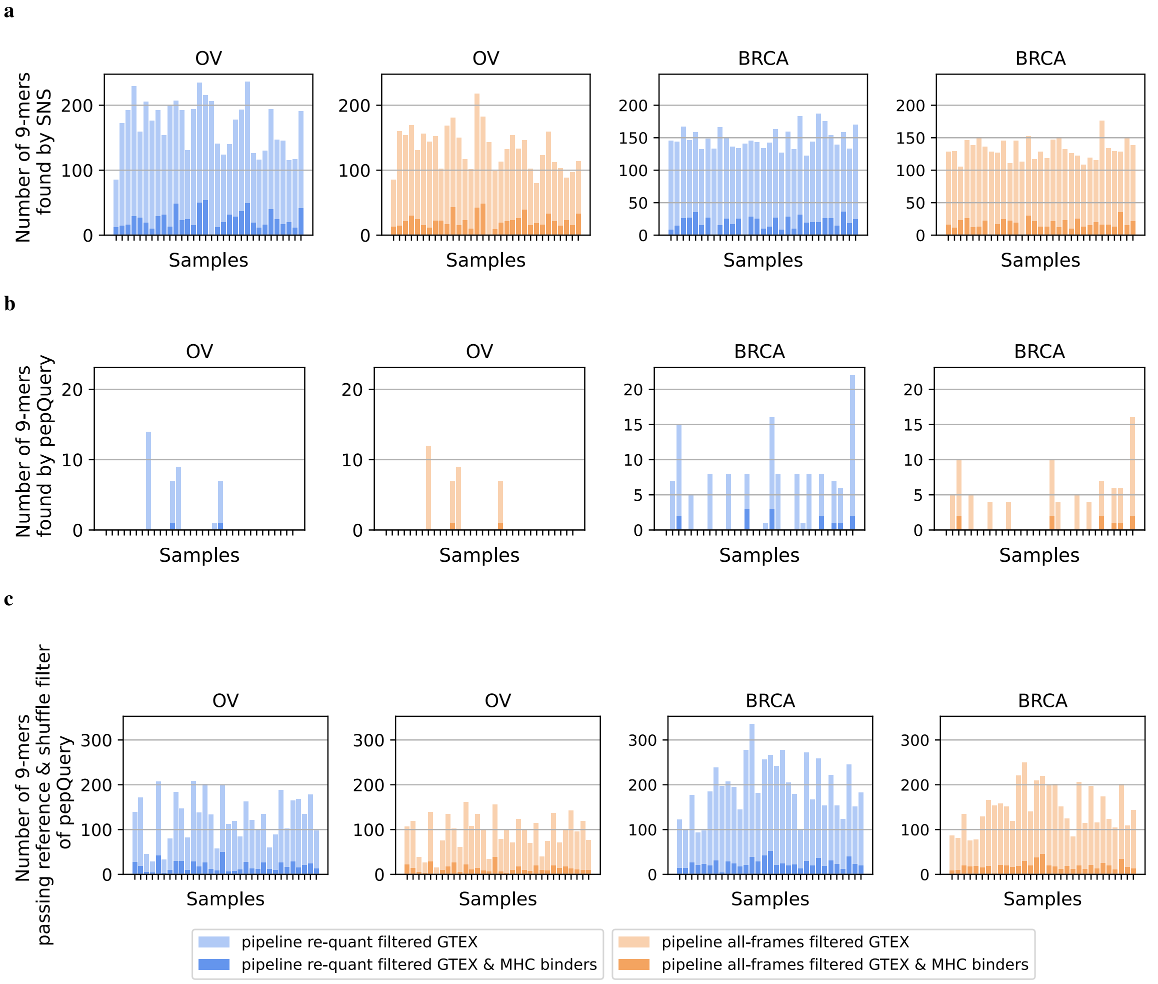

Fig. 2: **Number of 9-mers candidates confirmed with Mass Spectrometry (MS).** a: Number of 9-mers candidates validated with the *Subset-Neighbor-Search* method. b: Number of 9-mers candidates validated with the *PepQuery* method. c: P Number of 9-mers candidates validated with the *PepQuery* method when ignoring the last post-translational competition step. Blue: Output of pipeline *re-quant*. Orange: Output of pipeline *all-frames*. Lighter alpha: no restriction on MHC-binding. Darker alpha: restriction on MHC-binding.

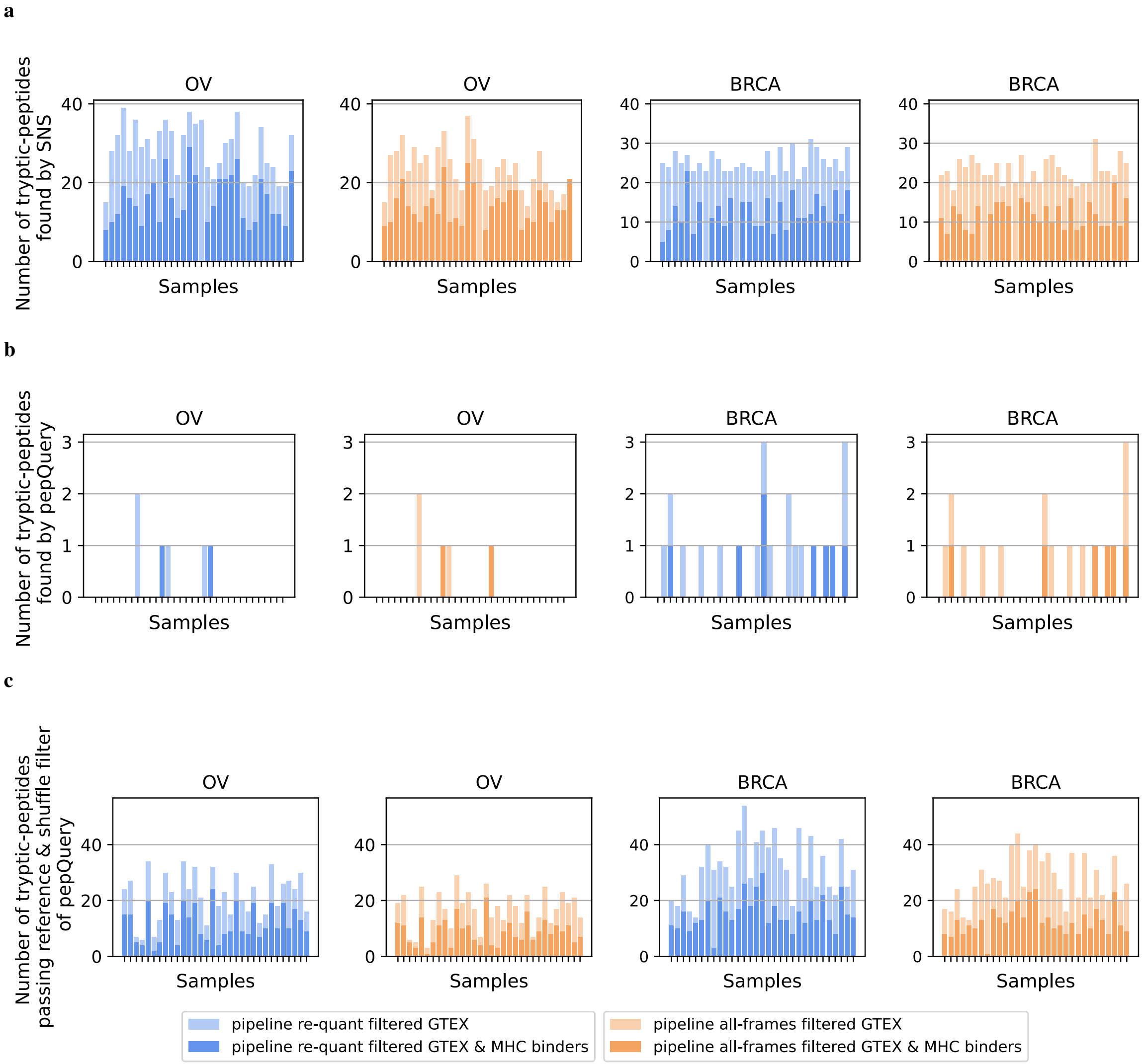

Fig. 3: **Number of tryptic-junction peptides candidates confirmed with Mass Spectrometry (MS).** a: Number of 9-mers candidates validated with the *Subset-Neighbor-Search* method b: Number of 9-mers candidates validated with the *PepQuery* method c: Number of 9-mers candidates validated with the *PepQuery* method when ignoring the last post-translational competition step. Blue: Output of pipeline *re-quant*. Orange: Output of pipeline *all-frames*. Lighter alpha: no restriction on MHC-binding. Darker alpha: restriction on MHC-binding.

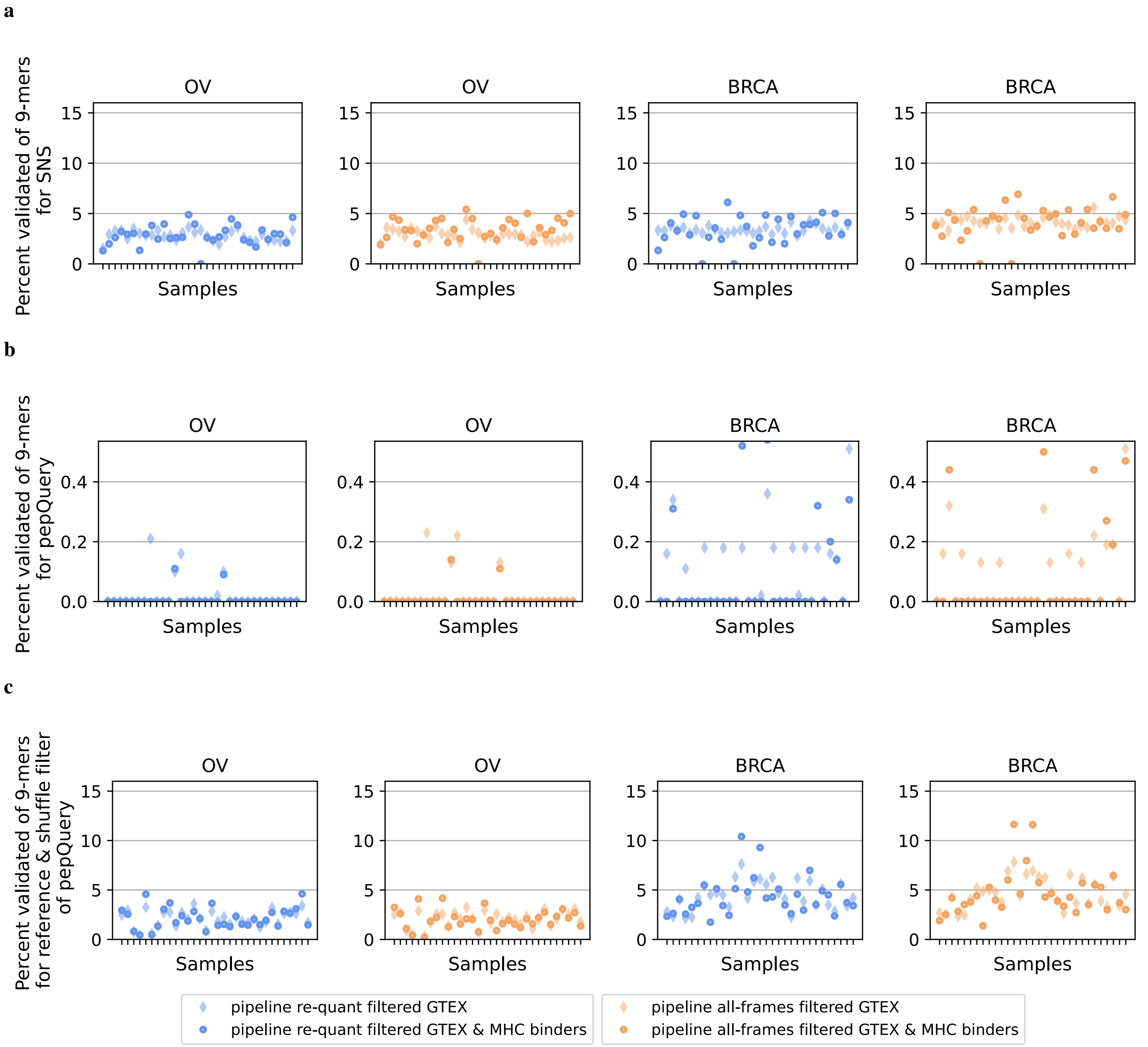

Fig. 4: **Percent validated 9-mers with Mass Spectrometry (MS).** a: Percent validated 9-mers with the *Subset-Neighbor-Search* method b: Percent validated 9-mers with the *PepQuery* method c: Percent validated 9-mers with the *PepQuery* method when ignoring the last post-translational competition step. Blue: Output of pipeline *re-quant*. Orange: Output of pipeline *all-frames*. Lighter alpha: no restriction on MHC-binding. Darker alpha: restriction on MHC-binding.

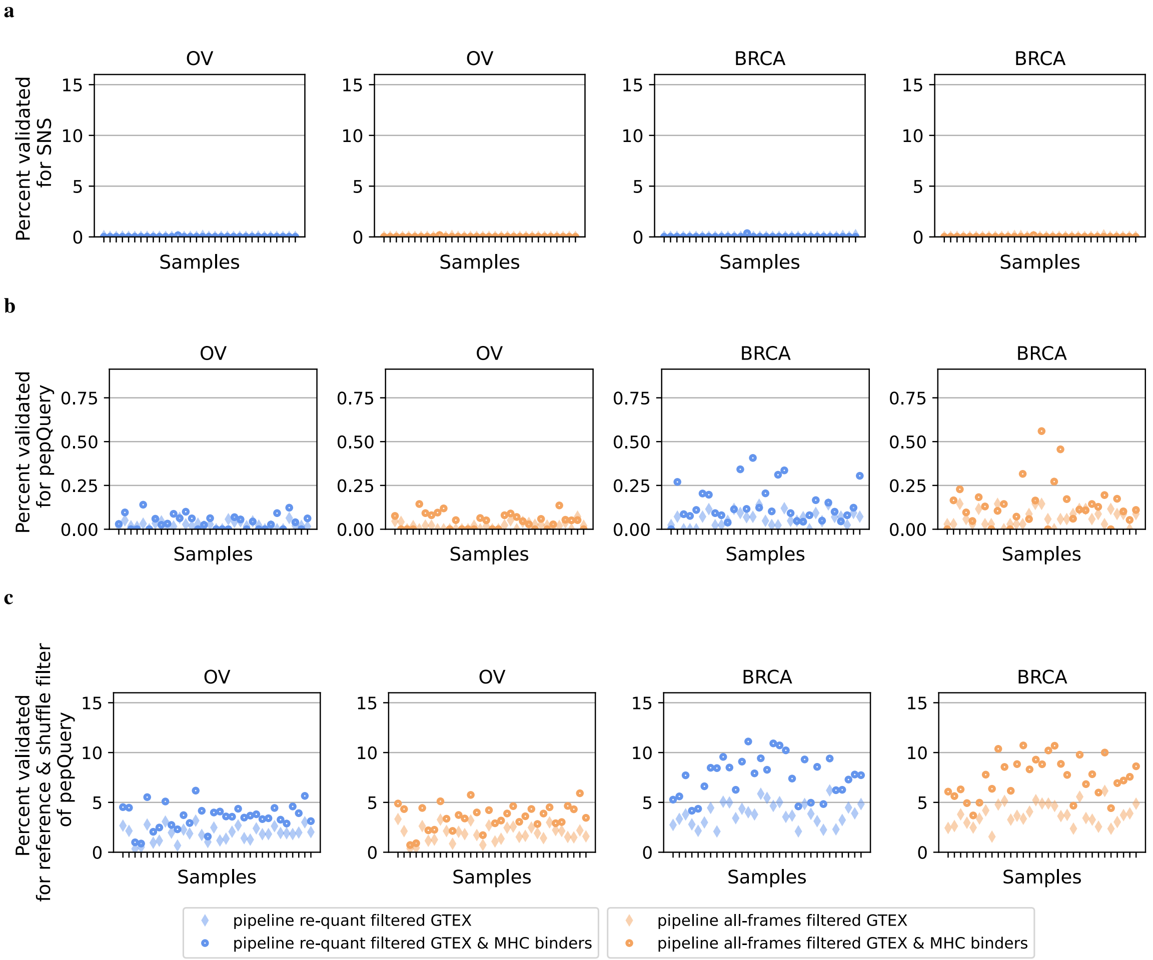

Fig. 5: **Percent of tryptic junction-peptides translated in a "wrong frame" validated with Mass Spectrometry (MS).** a: Percent of tryptic junction-peptides translated in a "wrong frame" validated with the *Subset-Neighbor-Search* method b: Percent of tryptic junction-peptides translated in a "wrong frame" validated with the *PepQuery* method c: Percent of tryptic junction-peptides translated in a "wrong frame" validated with the *PepQuery* method when ignoring the last post-translational competition step. Blue: Output of pipeline *re-quant*. Orange: Output of pipeline *all-frames*. Lighter alpha: no restriction on MHC-binding. Darker alpha: restriction on MHC-binding.

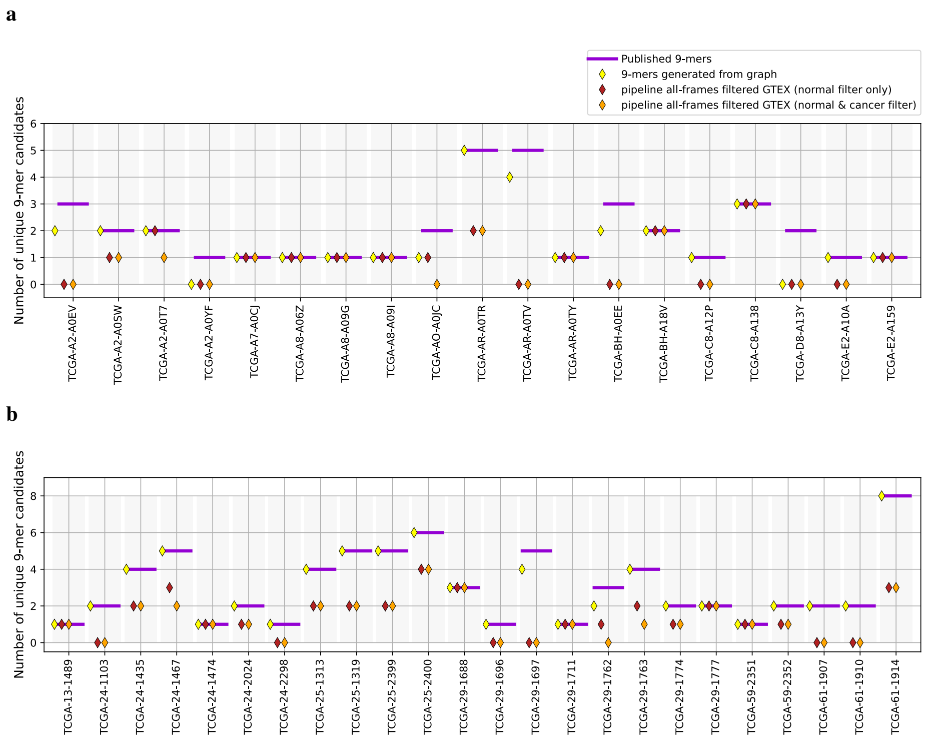

Fig. 6: **Recovery of previously published candidates (Kahles *et al.*, 2018) for the *all-frames* experimental setup.** The 9-mers are generated from the *merged* splicing graph, then 9-mers present in the GTEx cohort are filtered out. Finally expression and recurrence criteria are applied in the cancer cohorts. a: Recovery in BRCA samples b: Recovery in OV samples.

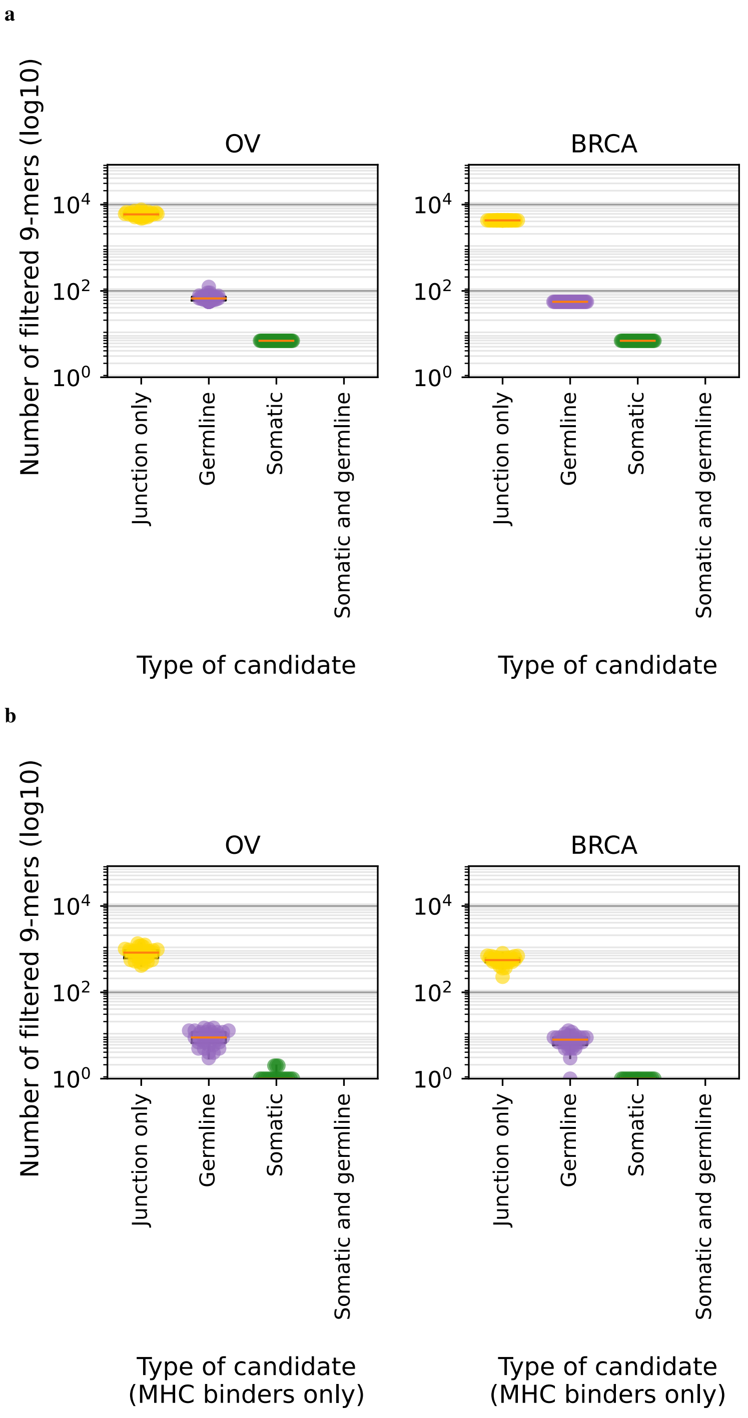

Fig. 7: **Number of tumor-associated 9-mers candidates separated by the variation type from which they were derived.** The candidate set is generated from the cancer sample's data after removing 9-mers present in GTEx and applying cancer support filtering criteria. Results are presented as the union across cohort samples. A: No restriction on MHC-binding. B: restriction on MHC-binding.

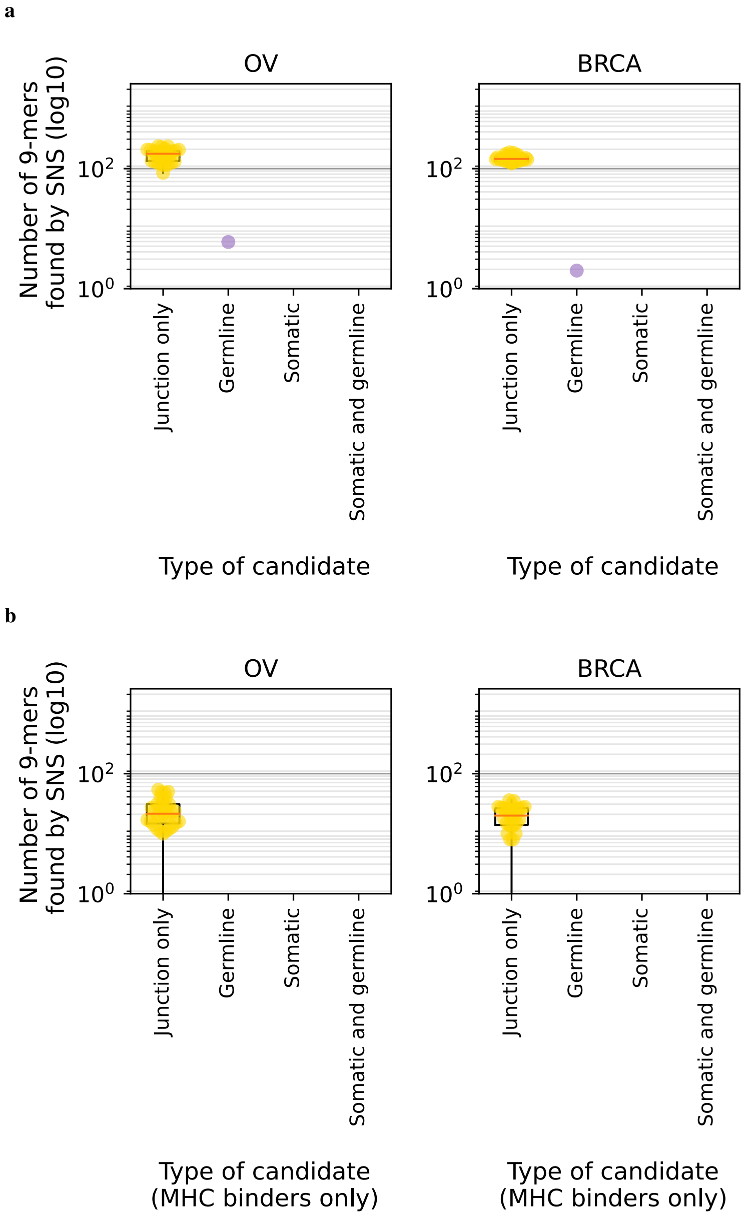

Fig. 8: **Number of tumor-associated 9-mers candidates validated with the *Subset-Neighbor-Search* method separated by the variation type from which they were derived.** The candidate set is generated from the cancer sample's data after removing 9-mers present in GTEx and applying cancer support filtering criteria. The MS validation is performed with the *Subset-Neighbor-Search* method. Results are presented as the union across cohort samples. A: No restriction on MHC-binding. B: restriction on MHC-binding.

b

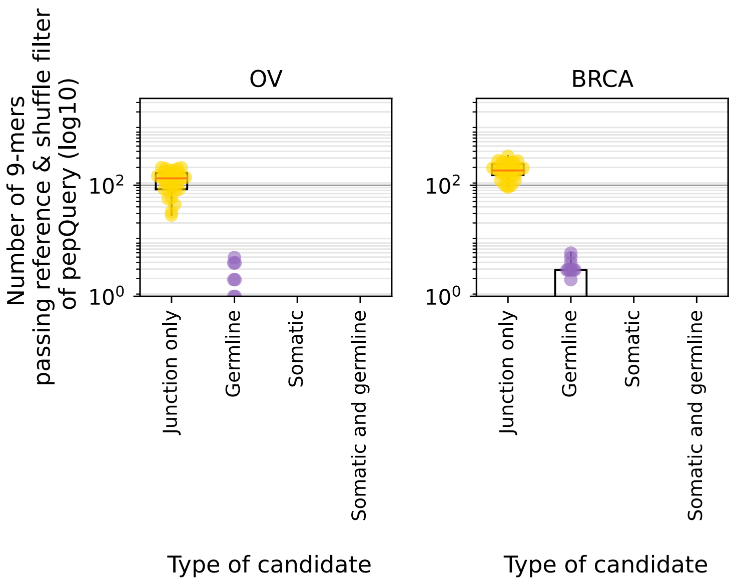

b

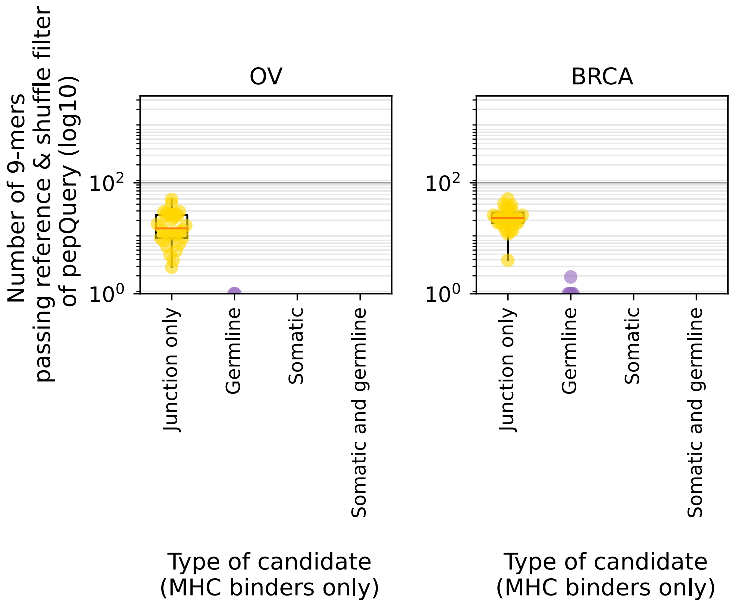

Fig. 9: **Number of tumor-associated 9-mers candidates validated with the *PepQuery** method separated by the variation type from which they were derived.** The candidate set is generated from the cancer sample's data after removing 9-mers present in GTEx and applying cancer support filtering criteria. (*) The MS validation is performed with *PepQuery* without the last post-translational competition step. Results are presented as the union across cohort samples. A: No restriction on MHC-binding. B: restriction on MHC-binding.

b

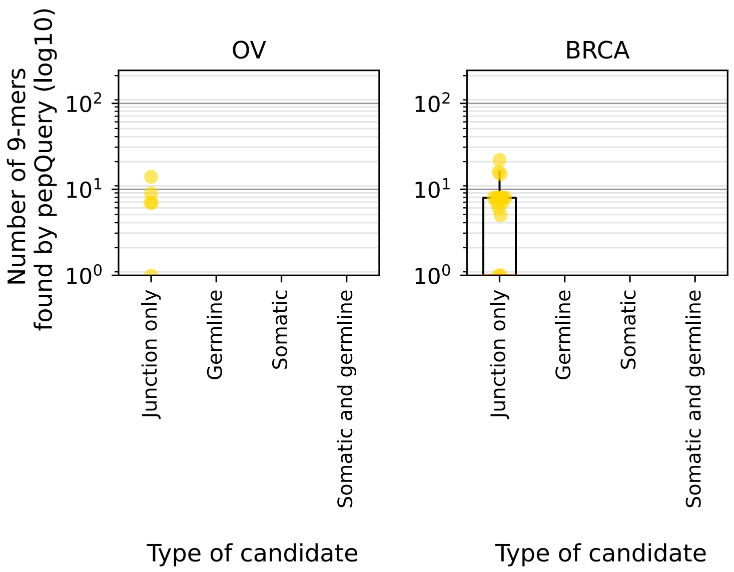

b

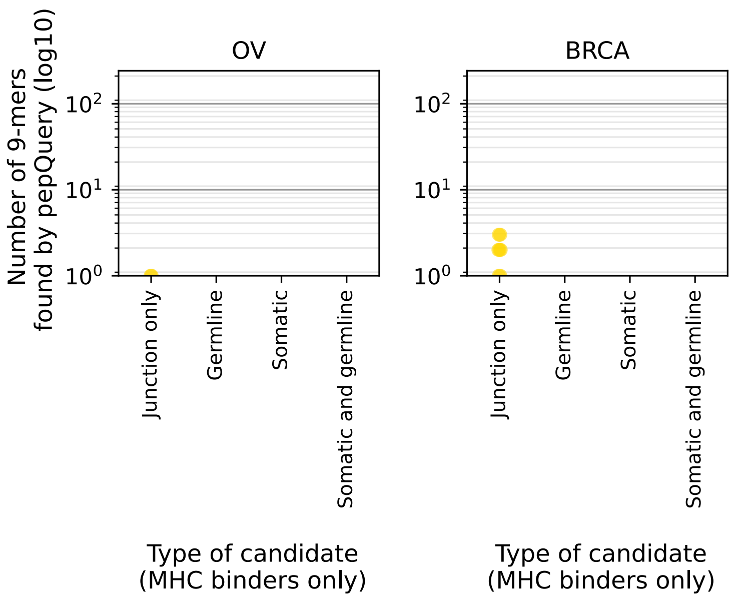

Fig. 10: **Number of tumor-associated 9-mers candidates validated with the *PepQuery** method separated by the variation type from which they were derived.** The candidate set is generated from the cancer sample's data after removing 9-mers present in GTEx and applying cancer support filtering criteria. (*) The MS validation is performed with *PepQuery* with the last post-translational competition step. Results are presented as the union across cohort samples. A: No restriction on MHC-binding. B: restriction on MHC-binding.
